# *In Situ* Kinetics of Solution-Phase Biomolecular Reactions and Interactions through Single-Molecule Displacement Statistics

**DOI:** 10.1021/acscentsci.6c00063

**Published:** 2026-05-05

**Authors:** Wan Li, Ke Xu

**Affiliations:** Department of Chemistry, 1438 University of California, Berkeley, Berkeley, California 94720, United States

## Abstract

We report a framework for quantifying the *in situ* reaction kinetics of bio­(macro)­molecules in the solution phase through single-molecule displacement statistics. Using single-molecule displacement/diffusivity mapping (SM*d*M), we measure the transient (0.6 ms) displacements of freely diffusing molecules in the wide field at ultrahigh throughput. Fitting the time-dependent displacement distributions of many molecules to a two-component diffusion model quantifies the reactant and product fractions based on molecular-weight differences, thereby enabling kinetic analysis of reaction progress at ∼1 s temporal resolution. Working with ∼100 pM fluorescently tagged reactant while varying the concentration of a second, unlabeled reactant over wide ranges, we determine second-order reaction rate constants for strain-promoted azide–alkyne cycloaddition and *N*-hydroxysuccinimide ester aminolysis as well as first-order hydrolysis side-reaction rate constants for the latter. For antibody binding kinetics, we quantify association and dissociation rate constants for a monoclonal antibody and unveil the gradual formation of cross-linked complexes/aggregates for a polyclonal antibody. Working with microliter-scale samples, our single-molecule-displacement-based approach provides intuitive readouts linked to the molecular size and is applicable to diverse reaction types and conditions.

## Graphical abstract

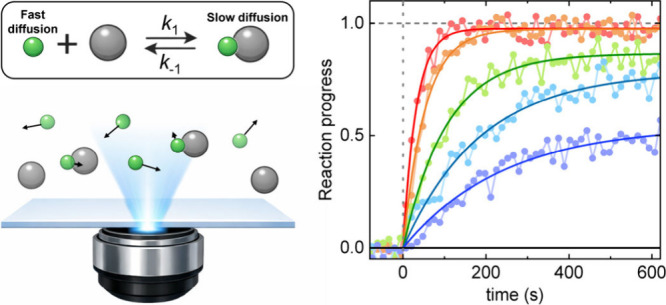

## Introduction

It is often difficult to monitor the reaction and interaction kinetics of bio­(macro)­molecules in the native aqueous solution phase. Common spectroscopy methods like NMR and IR disfavor the aqueous phase, suffer from limited sensitivity, and are poorly suited for *in situ* measurements. Moreover, each reaction requires the identification of a distinct signal that rises above the background to report the reaction progress, which is challenging given the often low (e.g., micromolar) concentrations of biomolecules in typical reactions.

For practically important bioconjugation reactions,^1–5^ fluorogenic reactions^5–8^ provide a possible path to evaluate reaction rates but are restricted to specific reactions and reactants and are difficult to generalize. UV–vis spectroscopy may monitor specific reactants or byproducts at high concentrations,^9^ but usually not the conjugated products, and is complicated by common protein and dye absorptions in the UV range. For antibody binding kinetics,^10^ typical detection methods such as surface plasmon resonance require surface immobilization, which may alter molecular interactions. The recent rise of microscale thermophoresis^11–13^ provides solution-phase binding assays but is unsuitable for kinetic analysis without special hardware modifications.^13,14^

Molecular diffusion provides a powerful means for particle sizing in the solution phase, thereby offering a window into molecular states and interactions.^15–20^ Sizing via traditional diffusion measurements such as fluorescence correlation spectroscopy (FCS) is challenged by numerous experimental factors, and it remains difficult to quantify absolute particle sizes.^19^ Single-molecule tracking^21–25^ quantifies molecular motion but is often limited to slow (diffusion coefficient *D* < ∼10 μm^2^/s) diffusion, unsuitable for free diffusion in the aqueous phase. Single-molecule trapping detects fast diffusion,^26^ but by monitoring only one molecule at a time, it does not report the ensemble reaction progress.

We recently introduced single-molecule displacement/diffusivity mapping (SM*d*M) to quantify molecular diffusion.^20,27^ By repeating tandem excitation pulses across paired camera frames, SM*d*M enables the high-throughput acquisition of transient (<1 ms) displacements of single molecules freely diffusing in the wide field. While initially developed for *D* spatial mapping,^27,28^ SM*d*M’s high-throughput single-molecule statistics also enables high-precision *D* quantification in homogeneous systems.^29–31^ Notably, in common buffers, the SM*d*M-measured *D* values for proteins and small solutes correlated scrupulously with the molecular weight over 0.6–600 kDa, matching the Young–Carroad–Bell (YCB) model^32^ without the need for adjustable parameters (Figure S1).^20,29,31,33^

Here we report a general framework for quantifying solution-phase reaction kinetics *in situ* using high-throughput single-molecule displacement statistics afforded by SM*d*M (Scheme 1). Starting with ∼100 pM of a fluorescently tagged reactant, we continuously perform SM*d*M to monitor how its diffusing behavior evolves over time, as a second, unlabeled reactant is added at varying concentrations to initiate a reaction to produce a product with a shifted molecular size. By temporally segmenting the SM*d*M-accumulated displacements of many single molecules to quantify the reactant and product fractions in the solution over time, we successfully monitor the kinetics of diverse reactions and interactions at ∼1 s temporal resolution.

**Scheme 1:**
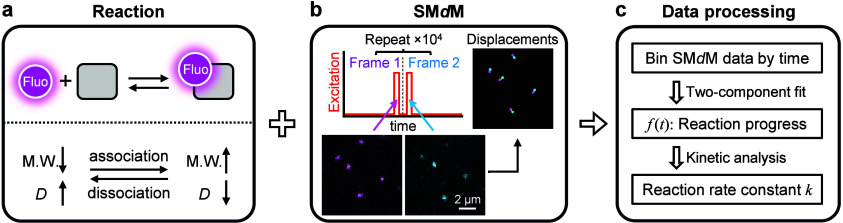
Monitoring Solution-Phase Reaction Kinetics *In Situ* through Single-Molecule Displacement Statistics^a^

a (a) Reactions/interactions alter the size of the fluorescently tagged moiety and consequently the measured diffusion coefficient *D*. (b) In SM*d*M, tandem excitation pulses are repeatedly applied across paired camera frames to acquire transient (*e.g.*, 0.6 ms) single-molecule displacements in the wide field with high throughputs. (c) Kinetic and two-component analyses of the SM*d*M-accumulated single-molecule displacements extract reactant/product fractions and reaction kinetics.

## Results and Discussion

### Two-Component Kinetic Analysis of SM*d*M Single-Molecule Displacements

We start by demonstrating how well SM*d*M quantifies molecular components in a mixture. To this end, we examine two noninteracting proteins, Cy3B-labeled ∼160 kDa donkey immunoglobulin G (IgG-Cy3B) and Cy3B-labeled 50 kDa donkey IgG Fab fragment (Fab-Cy3B). SM*d*M was performed for an ∼100 pM sample in ∼50 μL phosphate-buffered saline (PBS) using repeated excitation pulse pairs with a center-to-center separation of 0.6 ms across tandem camera frames (Scheme 1b),^20,27,29^ which we previously showed to enable efficient detection of single-molecule displacements in solution.^29^ At a framerate of 110 frames per second and with ∼12 single-molecule displacements detected across each frame pair, ∼700 single-molecule displacements were detected every second.

For pure IgG-Cy3B and Fab-Cy3B, the SM*d*M-accumulated single-molecule displacements were well-fitted with a single diffusion mode^20,27^ to yield *D* = 44.6 ± 0.5 and 73.3 ± 0.5 μm^2^/s (Figure 1a,b), respectively. The fitted *D* values are consistent with YCB predictions (Figure S1), whereas the relative uncertainties in *D* scale inversely with the square root of the number of single-molecule displacements detected.^29^ For IgG-Cy3B and Fab-Cy3B mixed at varied ratios, fitting the SM*d*M displacement distributions with a two-component model that utilized the above *D* values of pure IgG-Cy3B and Fab-Cy3B (Figure 1c; Methods) yielded fractions in agreement with the expected values (Figure 1d). Comparison of results with different fitting models further showed that the SM*d*M data correctly identify pure molecules and binary mixtures as one- and two-component systems, respectively (Figure S2).

**Figure 1:**
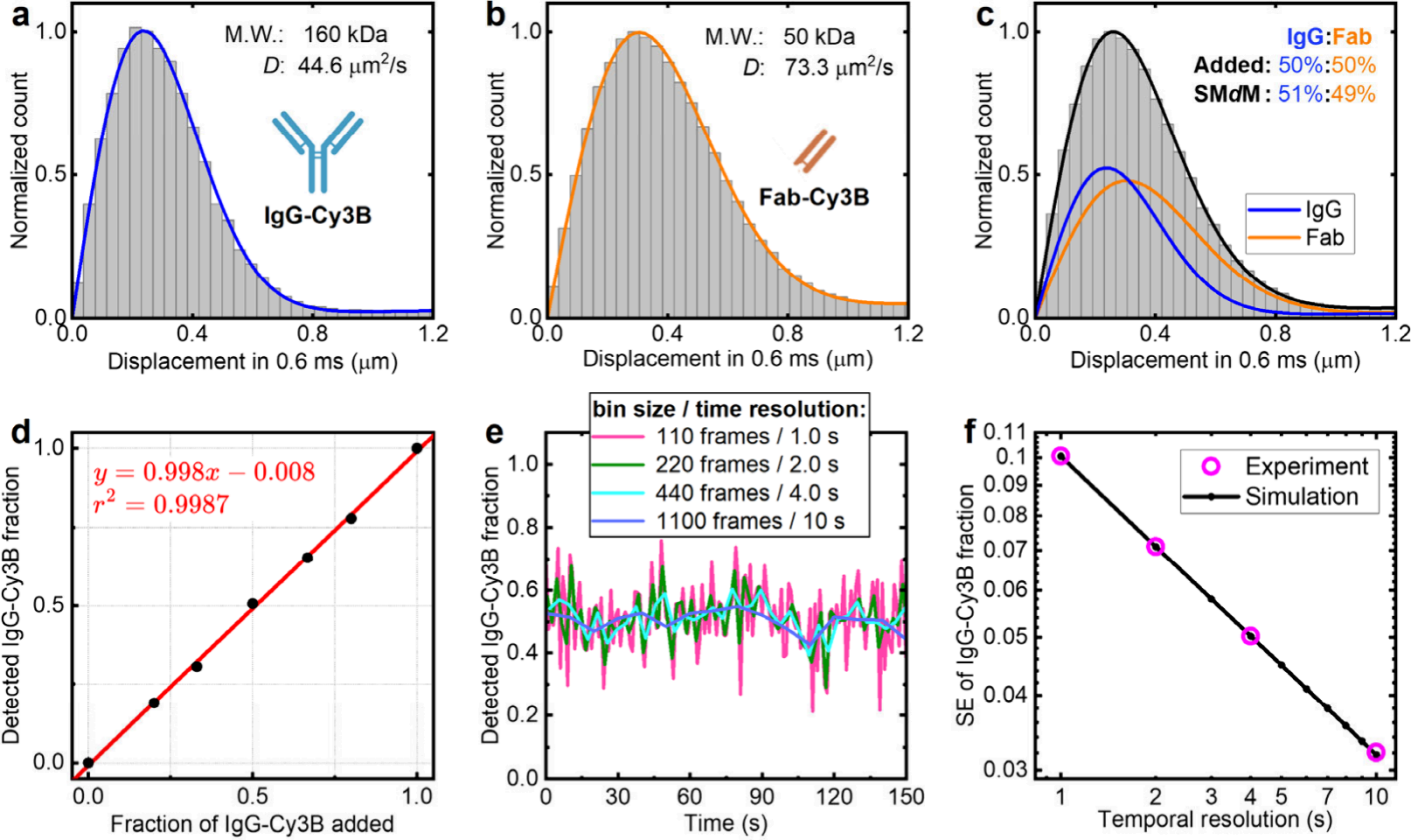
Two-component and kinetic analyses of SM*d*M single-molecule displacements. (a, b) Histograms: Distributions of SM*d*M-measured 0.6 ms single-molecule displacements of IgG-Cy3B (a) and Fab-Cy3B (b) in PBS. Curves: Fits to a single-component diffusion model, yielding *D* = 44.6 ± 0.5 and 73.3 ± 0.5 μm^2^/s, respectively. (c) Histogram: Distribution of SM*d*M-measured 0.6 ms single-molecule displacements for a 1:1 mixture of IgG-Cy3B and Fab-Cy3B in PBS. Curves: Fit to a two-component diffusion model (black) and its decomposition into IgG-Cy3B (blue) and Fab-Cy3B (orange) contributions, yielding 51% IgG-Cy3B. (d) Data points: Measured vs actual fractions of IgG-Cy3B for IgG-Cy3B and Fab-Cy3B mixed at varied ratios. Line: Linear fit to the data, with results given in the graph. (e) SM*d*M-extracted IgG-Cy3B fraction over time for the 1:1 mixture, obtained by segmenting the SM*d*M data by varied numbers of frames and thus temporal resolutions as marked in the legend. (f) Standard errors at different temporal resolutions from the experimental data in (e) (magenta) vs those from simulated data (black).

To demonstrate temporal resolution, we temporally segmented the SM*d*M-accumulated single-molecule displacements, so the displacements in each segment were individually fitted to the two-component model. The resulting IgG-Cy3B fraction values, e.g., for the 50% fraction sample (Figure 1e), centered around the expected values and showed reduced fluctuations with increased segment sizes. Standard errors of 0.050 and 0.032 were achieved for the determined fractions at 4 and 10 s temporal resolutions, respectively, scaling inversely with the square root of the temporal resolution, in agreement with our simulated results (Figure 1f). Simulation further predicted increased fraction uncertainties with reduced molecular-weight differences between the two components and *vice versa*, with 2-fold and 32-fold differences yielding standard errors of 0.066 and 0.018 at a 10 s temporal resolution, respectively (Figure S3). If we fluorescently tag the smaller reactant in a conjugation reaction, the molecular-weight difference between the SM*d*M-detected product and reactant is >2 and easily >10 (examples below), and their fractions in the mixture should be reliably quantified at 1–10 s temporal resolution.

### Reaction Kinetics of Strain-Promoted Azide–Alkyne Cycloaddition

For reaction kinetics, we start with the strain-promoted azide–alkyne cycloaddition (SPAAC), a popular copper-free click reaction that enables bioconjugation under physiological conditions.^3–5^ Commercial dibenzocyclooctyne-conjugated Alexa Fluor 647 (DBCO-AF647) was used as a typical SPAAC reactant. A soluble protein, ovalbumin (OVA), was functionalized with ∼2.7 azide groups per protein (Methods). Mixing DBCO-AF647 and OVA-N_3_ in PBS yielded the SPAAC product OVA-AF647 (Figure 2a). For the starting DBCO-AF647 and purified OVA-AF647 product, SM*d*M detected contrasting *D* values of 257 ± 5 and 71 ± 0.6 μm^2^/s, respectively, consistent with their 1.1 and 45 kDa molecular weights (Figure S1).

**Figure 2:**
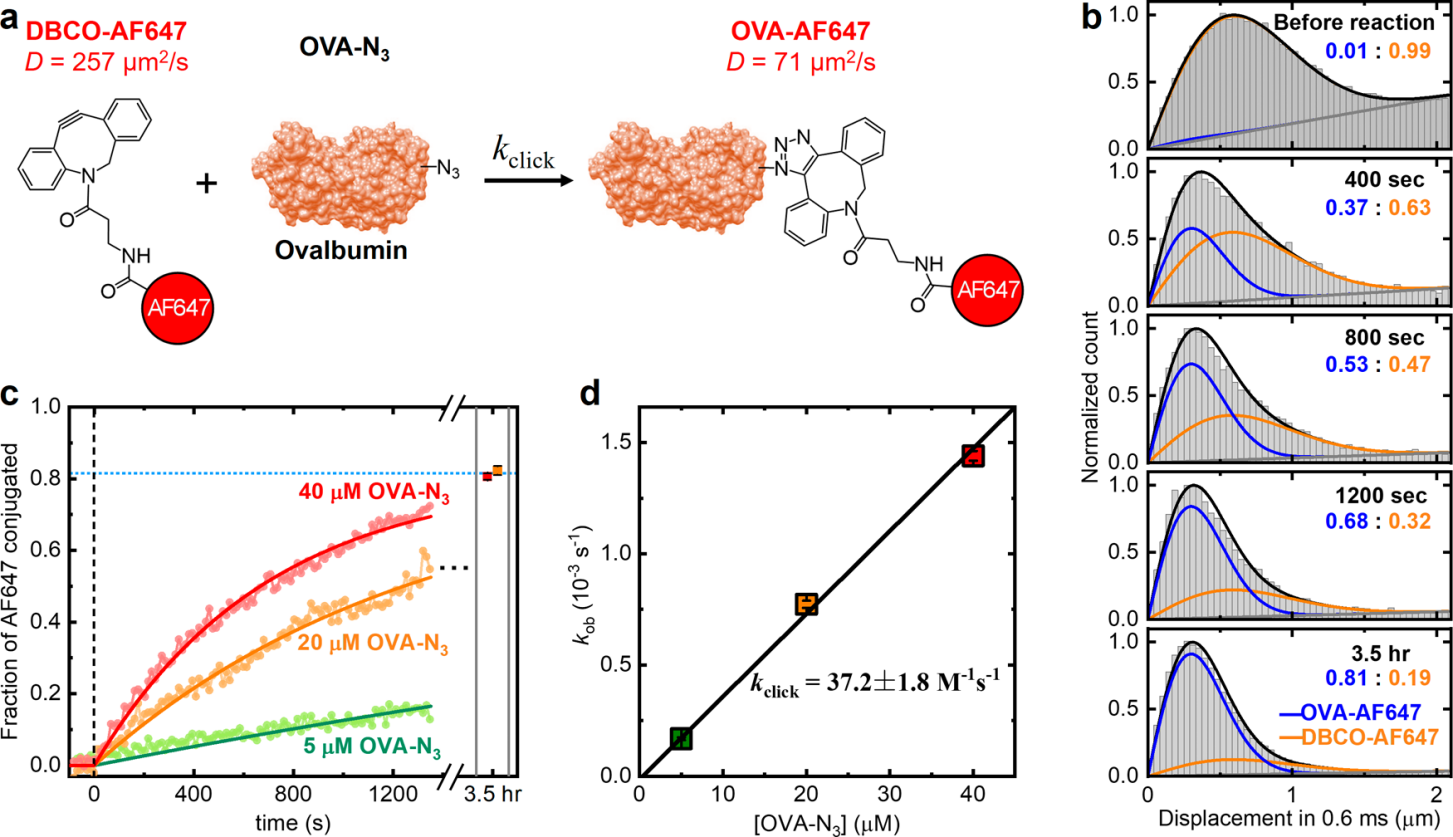
SM*d*M kinetic study of SPAAC. (a) Schematic: SPAAC between the 1.1 kDa DBCO-AF647 (*D* = 257 ± 5 μm^2^/s) and OVA-N_3_ generates the 45 kDa product OVA-AF647 (*D* = 71.0 ± 0.6 μm^2^/s). (b) Histograms: Distributions of SM*d*M-measured 0.6 ms single-molecule displacements for 100 pM DBCO-AF647 in PBS before and 400 s, 800 s, 1200 s, and 3.5 h after the addition of 40 μM OVA-N_3_. Curves: Fits to a two-component diffusion model (black) and their decomposition into OVA-AF647 (blue) and DBCO-AF647 (orange) contributions on top of single-molecule backgrounds (gray). The resulting OVA-AF647 and DBCO-AF647 fractions are labeled in each plot. (c) Dots: Reaction progress over time, presented as the fraction of OVA-AF647 of the detected AF647 molecules as extracted from two-component analysis of the SM*d*M data segmented at a 13.6 s temporal resolution, with 5, 20, and 40 μM OVA-N_3_ added at time 0. Curves: Fits to first-order reaction kinetics, from which the observed rate constants *k*_ob_ are determined. (d) Data points: *k*_ob_ from the fits in (c) plotted against the OVA-N_3_ concentration. Error bars: Standard error from the fits in (c). Line: linear fit to the data, yielding a second-order rate constant of *k*_click_ = 37.2 ± 1.8 M^–1^ s^–1^ from the slope.

Starting with 100 pM DBCO-AF647 in PBS, we ran SM*d*M continuously to monitor the diffusion behavior of AF647 in real time. As a control, adding unmodified OVA to the sample did not change the diffusion behavior of DBCO-AF647, suggesting no nonspecific interactions (Figure S4). In contrast, the addition of 40 μM (∼1.8 mg/mL) OVA-N_3_ led to a steady downshift in SM*d*M single-molecule displacements (Figure 2b), consistent with the gradual formation of the slower-diffusing OVA-AF647 through SPAAC (Figure 2a). As another control, the diffusivity of OVA-AF647 in PBS was unaffected by the addition of 40 μM OVA (Figure S4). Fitting the single-molecule displacements with a two-component model that utilized the above-measured *D* values of pure OVA-AF647 and DBCO-AF647 determined the fraction of the SPAAC product (Figure 2b). Temporally segmenting the single-molecule displacements at a 13.6 s resolution for individual two-component fitting thus yielded time-dependent reaction progress (Figure 2c). A positive correlation was observed between the reaction rate and the added OVA-N_3_ concentration (Figure 2c), as expected. Reactions with 20 and 40 μM OVA-N_3_ stabilized to similar final OVA-AF647 fractions of ∼0.82 after 3.5 h, suggesting reaction completion and ∼18% nonreactive dye in the starting DBCO-AF647 sample (Figure S5).

As the 100 pM DBCO-AF647 concentration used in SM*d*M is orders of magnitude lower than that of OVA-N_3_, the reaction was pseudo-first-order for DBCO-AF647. Consequently, at each OVA-N_3_ concentration, the reaction progress was reasonably fitted with first-order reaction kinetics (Figure 2c; Methods). Plotting the resulting first-order rate constants *k*_ob_ as a function of the OVA-N_3_ concentration showed a linear dependence through the origin (Figure 2d), consistent with a simple second-order reaction. From the slope of this dependence, we derived a second-order rate constant of *k*_click_ = 37.2 ± 1.8 M^–1^ s^–1^ (between DBCO-AF647 and the ∼2.7-azide-functionalized OVA-N_3_). This value is consistent with what we estimated from the consumption rate of an analogue DBCO molecule, using a > 10^6^-fold higher concentration for UV–vis spectroscopy (Figure S6).

### Codetermination of Aminolysis and Hydrolysis Rates of the *N*-Hydroxysuccinimide Ester

We next turn to the more complex reactions of the *N*-hydroxysuccinimide (NHS) ester. The amide-forming aminolysis between NHS esters and primary amines is among the most widely used bioconjugation methods.^1,34^ However, the aminolysis reaction is competed by hydrolysis, which reduces the conjugation yield (Figure 3a).

**Figure 3:**
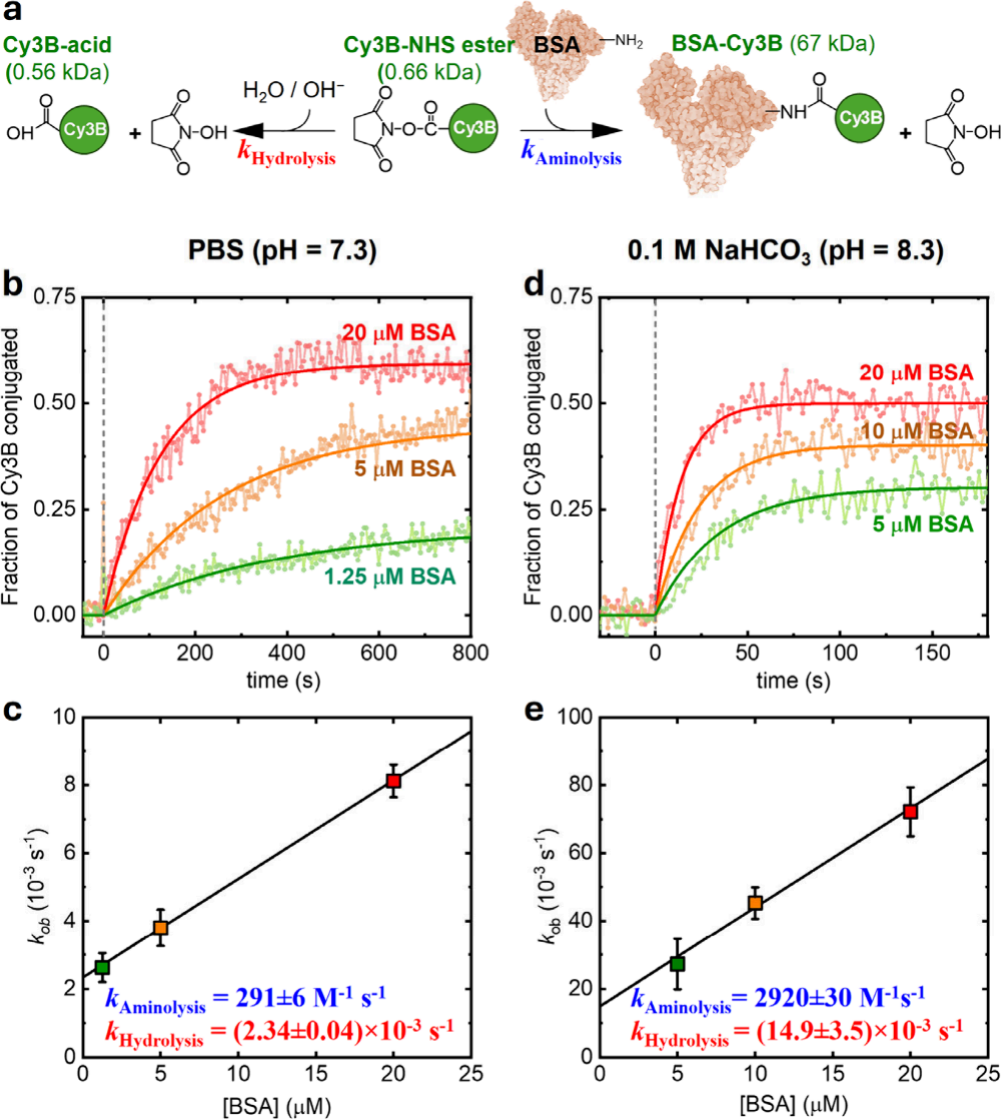
SM*d*M kinetic study of NHS-ester aminolysis and hydrolysis. (a) Schematic: Cy3B-NHS ester aminolysis with BSA to form BSA-Cy3B, accompanied by competing hydrolysis. (b) Dots: Reaction progresses over time in PBS, presented as the BSA-Cy3B fraction of detected Cy3B molecules from a two-component (BSA-Cy3B vs free Cy3B) analysis of the SM*d*M data segmented at a 4.5 s temporal resolution, with 1.25, 5, and 20 μM BSA added at time 0. Curves: fits to first-order reaction kinetics, from which the observed rate constants *k*_ob_ are determined. (c) Data points: *k*_ob_ from (b) plotted against the BSA concentration. Error bars: Standard error from the fits in (a). Line: linear fit to the data. The slope and the vertical intercept of the fit line give the aminolysis and hydrolysis rate constants, respectively, as *k*_Aminolysis_ = 291 ± 6 M^–1^ s^–1^ and *k*_Hydrolysis_ = (2.34 ± 0.04) × 10^–3^ s^–1^. (d, e) Similar to (b) and (c) but in 0.1 M NaHCO_3_, for which the SM*d*M data were segmented at a 1.8 s temporal resolution in (d) to capture the faster kinetics. The linear fit in (e) yields *k*_Aminolysis_ = 2920 ± 30 M^–1^ s^–1^ and *k*_Hydrolysis_ = (14.9 ± 3.5) × 10^–3^ s^–1^.

With SM*d*M, we monitored the reaction between Cy3B-NHS ester and the common soluble protein bovine serum albumin (BSA) (Figure 3a). Given the small difference in molecular weight between the reactant Cy3B-NHS ester (0.66 kDa) and its hydrolyzed product Cy3B-acid (0.56 kDa) when compared to the aminolysis product BSA-Cy3B (67 kDa), we fitted the SM*d*M single-molecule displacements with a two-component model (Figure S7) based on the separately determined *D* values of purified BSA-Cy3B at 58.5 ± 0.5 μm^2^/s and unbound Cy3B at 365 ± 9 μm^2^/s (Figure S1). The fraction of BSA-Cy3B from this two-component analysis thus represented reaction progress.

Starting with 100 pM of Cy3B-NHS ester in PBS (pH ∼ 7.3), we performed SM*d*M for 30 s and then added BSA. Concentration-dependent rises of the SM*d*M-determined BSA-Cy3B fraction were observed, which were well-resolved at a segment size of 500 frames (4.5 s temporal resolution) (Figure 3b). The conjugation proceeded faster than SPAAC above, but completed within minutes at lower yields. Lower BSA concentrations resulted in lower final yields, as expected due to competing hydrolysis of the Cy3B-NHS ester. Given the significant excess of BSA and water/hydroxide over the 100 pM Cy3B-NHS ester, both aminolysis and hydrolysis became pseudo-first-order for Cy3B-NHS ester in each experiment. Fitting the time-dependent reaction progresses to first-order kinetics yielded rate constants *k*_ob_ as sums of the aminolysis and hydrolysis rates (Methods). Plotting *k*_ob_ as a function of the BSA concentration (Figure 3c) showed a linear relationship, but now the fitted line did not pass through the origin, contrasting with our SPAAC results above. The finite *y*-intercept arises from the competing hydrolysis consumption of the NHS ester, which occurs even in the absence of the aminolysis substrate BSA. Simple derivations (Methods) showed that the slope of the fit gave the second-order rate constant of aminolysis, *k*_Aminolysis_ = 291 ± 6 M^–1^ s^–1^, whereas the *y*-intercept gave the first-order rate constant of hydrolysis, *k*_Hydrolysis_ = (2.34 ± 0.04) × 10^–3^ s^–1^.

We next compared results in 0.1 M NaHCO_3_ (pH ∼ 8.3), a typical experimental condition for NHS-ester-based bioconjugation.^34^ Significantly faster kinetics were noted, as quantified by fine-segmenting the SM*d*M data at a 1.8 s temporal resolution (Figure 3d). Similarly fitting *k*_ob_ for varied BSA concentrations (Figure 3e) revealed a 10-fold higher *k*_Aminolysis_ of 2920 ± 30 M^–1^ s^–1^. This result captures the expected pH dependence of the reaction:^34^ NHS esters react with unprotonated primary amines, the presence of which increases by ∼10-fold for lysine side chains (p*K*_a_ ≈ 10.5) as pH increases from 7.3 to 8.3. We also observed a 6-fold higher *k*_Hydrolysis_ at (14.9 ± 3.5) × 10^–3^ s^–1^. This result is consistent with the known acceleration of NHS-ester hydrolysis by hydroxide (OH^–^). Whereas our results in 0.1 M NaHCO_3_ over PBS identified a larger increase in *k*_Aminolysis_ than in *k*_Hydrolysis_, the initial 30 s period before BSA addition led to more hydrolysis and thus lower final conjugation yields.

### Quantifying the Binding Kinetics of a Monoclonal Antibody

We next examine antibody binding, which adds further layers of complication as noncovalent interactions permit reversible dissociation and nonstoichiometry. Here we applied SM*d*M to monitor how a 28.7 kDa green fluorescent protein (GFP) differentially interacts with anti-GFP monoclonal and polyclonal antibodies (mAb and pAb).

Figure 4a presents the SM*d*M-determined time-dependent *D* (left axis) for 1.2 nM GFP in PBS (with the addition of 1 mg/mL BSA to block nonspecific adsorption and interactions) and the corresponding apparent molecular weight converted based on the YCB model (right axis). Notably, the addition of 56 nM anti-GFP mAb (mouse IgG_2a_) led to a fast drop of *D* from 87.3 ± 1.2 μm^2^/s to a baseline of 44.9 ± 0.3 μm^2^/s within 1 min, translating to an increase in molecular weight from 25.9 ± 1.0 to 190 ± 4 kDa. Doubling the mAb concentration to 112 nM yielded similar final *D* values (44.9 ± 0.5 μm^2^/s) and hence converted molecular weights (190 ± 6 kDa) (Figure 4b). These results suggest a one-to-one GFP-mAb complex (∼184 kDa expected) in the limit of high mAb concentrations (Figure 4b), expected as the mAb binds to a single epitope in GFP.

**Figure 4:**
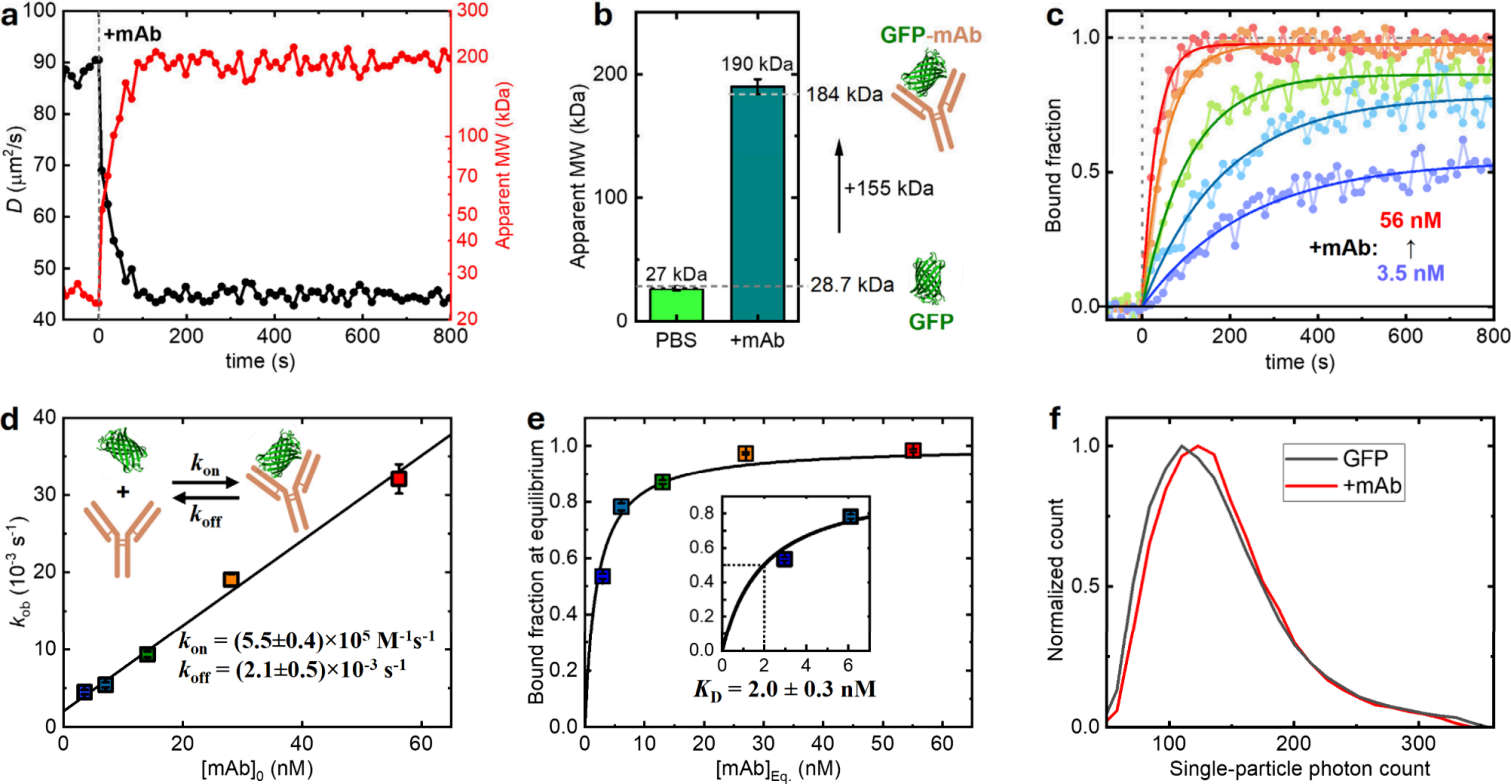
SM*d*M analysis of the binding kinetics of an anti-GFP mAb. (a) SM*d*M-determined time-dependent *D* (black, left axis) and YCB-converted apparent molecular weight (red, right axis) for 1.2 nM GFP in PBS (1 mg/mL BSA), with 56 nM anti-GFP mAb added at time 0. (b) Bars: Apparent molecular weights of GFP in PBS and in equilibrium with 112 nM mAb. Error bars: Standard deviations between 3 runs. Schematic: The molecular weight difference is consistent with one-to-one GFP-mAb conjugation. (c) Dots: time-dependent fraction of GFP-mAb in the detected GFP molecules, based on two-component (GFP-mAb vs free GFP) analysis of SM*d*M data segmented at a 13.6 s temporal resolution, with 3.5, 7.1, 14, 28, or 56 nM mAb added at time 0. Curves: Fits to first-order reaction kinetics, from which the observed rate constants *k*_ob_ are determined. (d) Data points: *k*_ob_ from (c) plotted against the mAb concentration. Error bars: Standard error from the fits in (c). Line: linear fit to the data, with the slope yielding the association rate constant *k*_on_ = (5.5 ± 0.4) × 10^5^ M^–1^ s^–1^ and the vertical intercept yielding the dissociation rate constant *k*_off_ = (2.1 ± 0.5) × 10^–3^ s^–1^. (e) Binding isotherm plotted as the fraction of GFP-mAb at equilibrium versus the concentration of free mAb at equilibrium, estimated by subtracting from the initial mAb concentrations the amount bound to the 1.2 nM GFP. Fitting to simple binding yields *K*_D_ = 2.0 ± 0.3 nM. (f) Distributions of single-particle photon counts in the SM*d*M data before (black) and 800 s after (red) adding 112 nM anti-GFP mAb, showing no substantial changes when compared to the addition of pAb (Figure 5c).

Having determined that the binding product is a one-to-one GFP-mAb complex of well-defined *D*, we applied a two-component analysis (Figure S8) using the above-determined *D* values of GFP-mAb and unbound GFP to extract the fraction of GFP-mAb as a readout of the binding progress (Figure 4c). As the mAb concentrations involved are generally higher than that of GFP, we again treated the process as pseudo-first order. In the presence of the opposing unbinding process, the fitted first-order *k*_ob_ at each mAb concentration is the sum of the association and dissociation rates (Methods). Consequently, plotting the resulting *k*_ob_ as a function of the mAb concentration showed a linear dependence (Figure 4d). Linear fit yielded a second-order association rate constant of *k*_on_ = (5.5 ± 0.4) × 10^5^ M^–1^ s^–1^ from the slope and a dissociation rate constant of *k*_off_ = (2.1 ± 0.5) × 10^–3^ s^–1^ from the *y*-intercept (Methods). Meanwhile, plotting the fitted GFP-mAb fraction at equilibrium as a function of the estimated concentration of free mAb generated a binding isotherm, with which we estimated a dissociation constant *K*_D_ = 2.0 ± 0.3 nM (Figure 4e). This value is reasonably close to what is expected from our *k*_on_ and *k*_off_ results as *K*_D_^′^ = *k*_off_/*k*_on_ = 3.8 ± 0.9 nM.

### Unveiling Contrasting Binding Kinetics and Complex Formation for a Polyclonal Antibody

We next study the interaction between GFP and an anti-GFP pAb (rabbit IgG). Interestingly, in contrast to the above mAb results, the SM*d*M-measured *D* after pAb addition did not stabilize but continued to decline over time in a concentration-dependent manner (Figure 5a). The apparent molecular weight converted from the observed *D* (Figure 5b) quickly rose above 1000 kDa at high pAb concentrations, with 125 nM pAb reaching >2000 kDa in 10 min. This result may be interpreted as that each GFP molecule is bound by multiple pAb IgG molecules targeting different epitopes. However, given the large IgG size, it appears difficult for each GFP molecule to accommodate more than a few IgG molecules. The gradually rising apparent size of >1000 kDa may be explained by considering that each pAb IgG molecule can further bind two GFP molecules through its two arms. The multivalency from both sides thus cross-links GFP and pAb into large complexes/clusters. Analogous cross-linking between antigens and pAbs is well-documented as the precipitin reaction at the macroscopic scale,^35^ while the very low antigen concentration here led to the gradual formation of nanoscale GFP-pAb complexes. Supporting this model, the brightness of the detected single particles in SM*d*M increased substantially with the addition of pAb, consistent with cross-linked complexes involving multiple GFP and IgG molecules (Figure 5c and inset). In comparison, no notable shifts were observed in single-particle brightness for the one-to-one binding of mAb to GFP (Figure 4f). Together, by revealing distinct binding modes and kinetics of mAb and pAb, SM*d*M demonstrated its versatility in elucidating protein–protein interactions.

**Figure 5:**
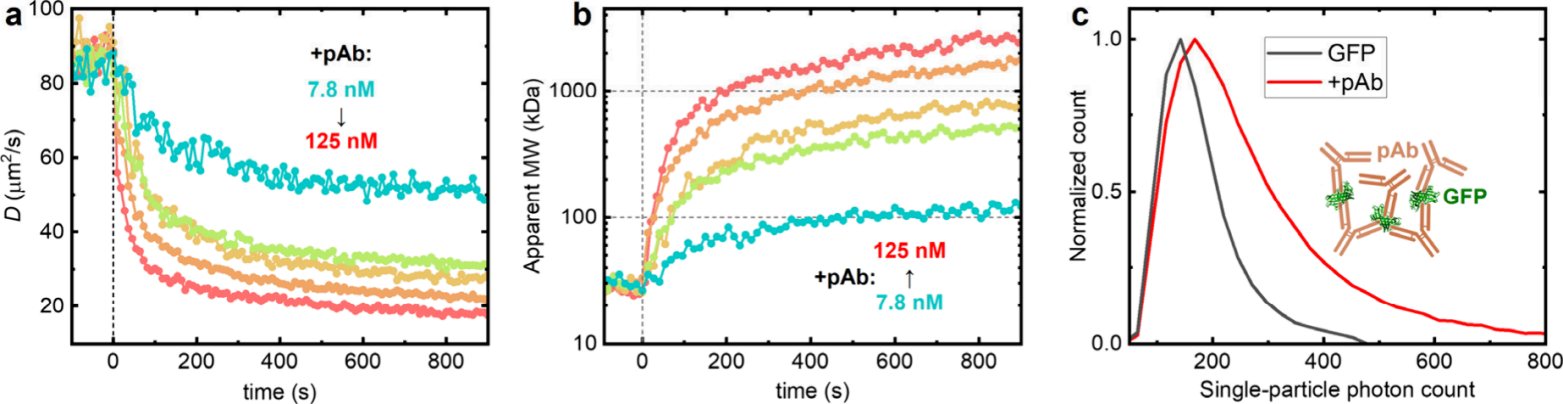
SM*d*M unveils a different binding mode for the anti-GFP pAb. (a) SM*d*M-determined time-dependent *D* for 1.2 nM GFP in PBS (1 mg/mL BSA) with 7.8, 15.6, 31.3, 62.5, and 125 nM anti-GFP pAb added at time 0. (b) Corresponding apparent molecular weight over time converted from the *D* values in (a) based on the YCB model. (c) Distributions of single-particle photon counts in the SM*d*M data, before (black) and 800 s after (red) adding 125 nM anti-GFP pAb. Inset schematic: Multivalency of both GFP and pAb leads to cross-linking and the formation of large complexes.

## Conclusions

Harnessing the high-throughput single-molecule displacement statistics of SM*d*M, we have demonstrated a general framework for monitoring *in situ* biomolecular reaction/interaction kinetics in the solution phase at ∼1 s temporal resolution. Requiring only ∼100 pM of a fluorescently tagged reactant in microliter-scale samples, this approach allows unlabeled reactants to be applied at arbitrary concentrations and enables straightforward kinetic analysis under pseudo-first-order conditions. Using this framework, we quantitatively extracted reaction rate constants for diverse reactions and interactions, including side and reverse reactions, and further unveiled the gradual formation of large, cross-linked complexes during pAb binding.

This work addresses the long-standing challenge in tracking biomolecular reaction/interaction kinetics in the native aqueous phase, where conventional spectroscopic and surface-based methods are often limited by sensitivity, selectivity, or perturbation of native interactions. By exploiting molecular diffusion as an intrinsic, reaction-agnostic physical readout of molecular size, our SM*d*M-based framework obviates the need for reaction-specific spectroscopic signals and is applicable to diverse reaction types and conditions, provided that one of the reactants is fluorescently labeled and undergoes substantial changes in molecular size in the reaction. The diffusion-based sizing, quantitatively mapped to molecular weights and reactant/product fractions, provides intuitive access to reaction progress. While FCS also detects diffusion changes, it provides limited sensitivity and remains difficult to quantify absolute diffusion coefficients and molecular weights (Figure S9).

The microliter-scale sample requirement and the demonstrated ability to resolve diverse reaction rates, such as the 10-fold difference we observed for the Cy3B-NHS-ester reaction under varied pH, highlight the potential of this approach for elucidating reaction mechanisms and optimizing reaction conditions. Extending this framework to broader classes of reactions and more complex environments (beyond our demonstrated antibody-binding assay in the presence of BSA), and further leveraging the spatial information inherent to SM*d*M to generate maps of local reactivity, represent promising future directions.

## Supplementary Material





## Notes

Materials and methods; SM*d*M-measured *D* versus molecular weight; expected uncertainties in fraction determination for varied molecular-weight differences; control experiments; HPLC analysis of DBCO-AF647; comparisons with UV–vis spectroscopy and FCS measurements; additional examples of two-component fitting of single-molecule displacements (PDF)
Transparent Peer Review report available (PDF)
