# Supplementary material for: In Situ Kinetics of Solution-Phase Biomolecular Reactions and Interactions through Single-Molecule Displacement Statistics
Source: ACS Cent Sci. 2026 May 5;12(5):712–8. doi: 10.1021/acscentsci.6c00063 (PMC13220185; doi:10.1021/acscentsci.6c00063)
Supplement: Supplementary file 1 [file oc6c00063_si_001.pdf]

Supporting Information for Publication

In Situ Kinetics of Solution-Phase Biomolecular Reactions and Interactions Through Single-Molecule Displacement Statistics

*Wan Li, Ke Xu**

Department of Chemistry, University of California, Berkeley, Berkeley, California 94720, United States

* Corresponding author: xuk@berkeley.edu (K.X.)

Materials and Methods

Sample preparation. Proteins used in this work are listed in **Table S1**. Donkey IgG (Jackson ImmunoResearch, #715-005-151) and Fab fragment of donkey IgG (Jackson ImmunoResearch, #711-007-003) were labeled with Cy3B-NHS (*N*-hydroxysuccinimidyl) ester (Cytiva PA63101) in 0.1 M $NaHCO_3$. Unconjugated dye was removed through >4 rounds of filtration using Amicon centrifugal filters (Millipore UFC5050 and UFC5030) until no detectable free dye remained in the filtrate. The degrees of labeling were 0.30 Cy3B dyes per IgG protein and 0.46 Cy3B dyes per Fab protein, respectively, as determined by a Nanodrop 2000c spectrophotometer (Thermo Fisher). Given the low labeling of both proteins, we expect that the fluorescently detected IgG-Cy3B and Fab-Cy3B in our experiments were singly labeled, while the unlabeled proteins remained undetected.

Azidated ovalbumin (OVA-N_3) was prepared by reacting ovalbumin from chicken egg white (Sigma #A5503) with 16× excess azidobutyric acid NHS ester (Lumiprobe, #53720) in 0.1 M $NaHCO_3$. The OVA-N_3 product was purified through Amicon Ultra centrifugal filters (Millipore UFC5010) 4 times. To estimate the degree of labeling, the purified OVA-N_3 was reacted with 20× excess of DBCO-AF647 (Click Chemistry Tools, #1302-1) for 24 hr at room temperature. The resulting OVA-AF647 was purified using Amicon centrifugal filters 4 times until no detectable AF647 was found in the filtrate. The molar ratio of AF647 to OVA was determined using a Nanodrop 2000c spectrophotometer to be 2.7. Assuming all functionalized azide groups had been conjugated, the average number of azide groups per OVA-N_3 molecule was estimated as 2.7.

Device preparation. Rectangular (20 mm × 50 mm) #1.5 glass coverslips were treated with a 3:1 H_2SO_4/H_2O_2 solution for 30 min, and then further activated with 1 M NaOH for 30 min to maximize surface hydroxyl groups. The freshly activated coverslips were thoroughly rinsed with Milli-Q water, dried with nitrogen gas, and baked at 60 °C for 5 min. Subsequently, the coverslip was coated with 80 μL of 25 mg/mL mPEG silane (5 kDa, Laysan Bio, MPEG-SIL-5000-1GR) in DMSO,[1] incubated at 60 °C for 1 hr, and then rinsed with Milli-Q water. To assemble the imaging chambers, plastic tubes (cut from PCR tubes, ~5 mm in diameter) were mounted onto the PEG-functionalized coverslip using epoxy.[2]

SM*d*M data collection. SM*d*M was performed at room temperature (23±1 °C) using a Nikon Ti-E inverted fluorescent microscope, as described previously,[3,4] 0.1-1 nM of fluorescent sample/reactant in PBS (Gibco 14190), or in another medium as indicated, was added to the above imaging device for a typical volume of 50-200 μL. Lasers of 488-nm (for GFP), 560-nm (for Cy3B), and 647-nm (for AF647) wavelengths were focused onto the back focal plane of an oil immersion objective lens (CFI Plan Apochromat Lambda 100×, NA = 1.45), and entered the sample slightly below the critical angle of the glass-solution interface to illuminate a wide field a few micrometers into the solution. The focal plane was maintained ~3 μm

above the glass-solution interface to record single molecules that diffused into it stochastically. A multifunction I/O board (PCI-6733, National Instruments) synchronized the timing signal from the EM-CCD camera (Andor iXon Ultra 897) with laser modulation, so that paired laser pulses of τ = 313 μs duration were repeatedly applied across tandem camera frames (**Scheme 1b**) at a center-to-center separation of Δt = 626 μs, as verified using a GW Instek GDS-1054B oscilloscope. The EM-CCD ran continuously at 110 frames per second to record single-molecule images in the wide field. In a typical run, we started SM*d*M by recording ~30 s of the fluorescently tagged reactant. The unlabeled other reactant was then added and mixed into the sample as SM*d*M continued to run for another 800-1,500 s to monitor reaction progress.

SM*d*M data analysis. SM*d*M analysis followed our previous approaches.[3,4] Single-molecule images were first localized in all frames using the GDSC SMLM analysis package.[5] For each pair of tandem frames, single-molecule localizations identified in the second frame were each searched for a matching localization in the first frame within a cutoff radius R of ~2 μm. Two-dimensional displacements r were calculated for the matched localizations. These single-molecule displacements over the fixed time interval Δt were then fitted to the single- or two-component probability models below.

For single-component analysis, the probability model was based on a two-dimensional random walk:[3]

$$P(r) = \frac{2r}{a}\exp(-\frac{r^2}{a}) + br \quad \textbf{(Eqn. 1)}$$

where $a = 4D\Delta t$ with D being the diffusion coefficient, and b accounts for a uniform background due to extraneous molecules that randomly diffuse into the search radius during Δt, as validated previously.[3] Fitting the experimental r to this model through maximum likelihood estimation (MLE) yielded a, which was then converted to D using the fixed Δt from the experiment.

Two-component analysis used an extended probability model:[1]

$$P(r) = F_1\frac{2r}{a_1}\exp(-\frac{r^2}{a_1}) + F_2\frac{2r}{a_2}\exp(-\frac{r^2}{a_2}) + br \quad \textbf{(Eqn. 2)}$$

where F_1 and $F_2 = 1 - F_1$ are the fractions of the two diffusing components (*e.g.*, the product and the reactant), $a_1 = 4D_1\Delta t$ and $a_2 = 4D_2\Delta t$ account for the diffusion coefficients D_1 and D_2 of the two components, and b accounts for a uniform single-molecule background. For reaction-kinetics analysis, we started by determining the D_1 and D_2 values from separate SM*d*M measurements of the pure product and pure reactant using **Eqn. 1**. The determined D_1 and D_2 (and thus a_1 and a_2) values were then used to fit the single-molecule displacements measured on mixtures to **Eqn. 2** to obtain F_1 and $F_2 = 1 - F_1$.

Analysis of reaction kinetics. For kinetic analysis, the SM*d*M data were first segmented by frames using segment sizes of 200–1500 frames, corresponding to 1.8–13.6 s, depending on the reaction rate and thus the desired temporal resolution. Two-component analysis (above) was then separately applied to the single-molecule displacements in each segment to extract F_1 (fraction of the product) and $F_2 = (1 - F_1)$ (fraction of the reactant) as a function of time t. In most cases, we plotted F_1 versus time, which typically started from 0 and gradually rose to a final value of $F_{1,eq} < 1$ (**Figures 2c, 3bd, 4c**).

The $F_1(t)$ results were next analyzed based on basic reaction kinetics, *e.g.* those discussed in typical textbooks.[6] For reactions obeying (pseudo)first-order reaction kinetics, $F_1(t)$ was fitted to the single-exponential equation below to obtain the observed rate constant (k_{ob}) and/or half-time $t_{½} = \ln2/k_{ob}$:

$$F_1(t) = F_{1,eq}(1 - e^{-k_{ob}t}) \quad \textbf{(Eqn. 3)}$$

where $F_{1,eq}$, the final fraction of the product at equilibrium, was either (i) treated as a free parameter to be determined concurrently with k_{ob} during fitting, or (ii) in the case of SPAAC reaction, fixed as 0.815 determined from SM*d*M measurements of fully reacted samples after 3.5 hours, interpreted as 18.5% non-reacting dye in the starting DBCO-AF647 sample. See below how **Eqn. 3** was applied in each scenario.

For the SPAAC reaction DBCO-AF647 + OVA-N_3 → OVA-AF647, as the 100 pM DBCO-AF647 concentration in SM*d*M is orders of magnitude lower than that of OVA-N_3, negligible OVA-N_3 is consumed during the reaction. Consequently, for each given starting OVA-N_3 concentration, [OVA-N_3] is effectively a constant. The reaction rate $r = k_{click}$[OVA-N_3][DBCO-AF647] is thus pseudo-first order for DBCO-AF647 as $r = k_{ob}$[DBCO-AF647] with $k_{ob} = k_{click}$[OVA-N_3]. $F_1(t)$ obtained at each OVA-N_3 concentration was separately fitted to **Eqn. 3** (**Figure 2c**) to obtain k_{ob} with $F_{1,eq}$ fixed as 0.815. Given k_{ob} = k_{click}[OVA-N_3], plotting the fitted k_{ob} as a function of the OVA-N_3 concentration showed a linear trend, for which a linear fit gave the second-order k_{click} from the slope (**Figure 2d**).

For NHS ester, we consider the branching reactions of aminolysis and hydrolysis. For aminolysis, given the significant excess of BSA over the 100 pM Cy3B-NHS ester, the reaction rate $r_{Aminolysis}$ = $k_{Aminolysis}$[BSA][Cy3B-NHS ester] is pseudo-first order for Cy3B-NHS ester as $r_{Aminolysis}$ = $k_{ob,Aminolysis}$[Cy3B-NHS ester] with $k_{ob,Aminolysis}$ = $k_{Aminolysis}$[BSA]. For hydrolysis, the reaction may be considered first-order for Cy3B-NHS ester with reaction rate $r_{Hydrolysis}$ = $k_{Hydrolysis}$[Cy3B-NHS ester]. Alternatively, if we consider that the hydrolysis is driven by hydroxide (OH^-), the 100 pM Cy3B-NHS ester concentration is substantially lower than the ~10^{-6} M [OH^-] and the buffer capacity, so the reaction is pseudo-first order for Cy3B-NHS ester with an apparent first-order $k_{Hydrolysis}$. The total reaction rate in terms of the consumption of Cy3B-NHS ester is thus $r = k_{eff}$[Cy3B-NHS ester] with $k_{eff} = k_{ob,Aminolysis}$ + $k_{Hydrolysis}$. The evolution of the fraction of the aminolysis product is thus:[6] $F_1(t) = \frac{k_{ob,Aminolysis}}{k_{eff}} F_2(0)(1 - e^{-k_{eff}t})$.

Here $F_2(0)$ is the fraction of active Cy3B-NHS ester at time 0, which is reduced from 1 by non-reacting dye, including the fraction hydrolyzed in the initial ~30-s period of our experiments before time 0, during which BSA was not added. This equation is identical to **Eqn. 3** above with $F_{1,\text{eq}} = \frac{k_{\text{ob,Aminolysis}}}{k_{\text{eff}}} F_2(0)$ and $k_{\text{ob}} = k_{\text{eff}}$. Thus, fitting the experimental $F_1(t)$ at each BSA concentration to **Eqn. 3** (**Figure 3bd**) yielded k_{ob} that corresponded to $k_{\text{eff}} = k_{\text{ob,Aminolysis}} + k_{\text{Hydrolysis}} = k_{\text{Aminolysis}}[\text{BSA}] + k_{\text{Hydrolysis}}$. Plotting the fitted k_{ob} as a function of the BSA concentration thus showed a linear trend, to which a linear fit gave the second-order $k_{\text{Aminolysis}}$ from the slope and the first-order $k_{\text{Hydrolysis}}$ from the y-intercept (**Figure 3ce**).

For GFP-mAb interaction, we consider the opposing reactions of association and dissociation, $\text{GFP} + \text{mAb} \rightleftarrows \text{GFP-mAb}$. Under the condition that the mAb concentrations are substantially higher than that of GFP, the association process with $r_{\text{on}} = k_{\text{on}}[\text{mAb}][\text{GFP}]$ is pseudo-first order for GFP as $r_{\text{on}} = k_{\text{ob,on}}[\text{GFP}]$ with $k_{\text{ob,on}} = k_{\text{on}}[\text{mAb}]$. The dissociation process is first-order for GFP-mAb with $r_{\text{off}} = k_{\text{off}}[\text{GFP-mAb}]$. The evolution of these two opposing first-order processes gives:[6] $[\text{GFP}] = ([\text{GFP}]_0 - [\text{GFP}]_{\text{eq}})e^{-(k_{\text{ob,on}}+k_{\text{off}})t} + [\text{GFP}]_{\text{eq}}$. Dividing off the initial GFP concentration $[\text{GFP}]_0$ yields: $F_2 = (1 - F_{2,\text{eq}})e^{-(k_{\text{ob,on}}+k_{\text{off}})t} + F_{2,\text{eq}}$. If monitoring the fraction of GFP-mAb: $F_1 = 1 - F_2$ $= (1 - F_{2,\text{eq}}) - (1 - F_{2,\text{eq}})e^{-(k_{\text{ob,on}}+k_{\text{off}})t} = F_{1,\text{eq}} - F_{1,\text{eq}}e^{-(k_{\text{ob,on}}+k_{\text{off}})t} = F_{1,\text{eq}}(1 - e^{-(k_{\text{ob,on}}+k_{\text{off}})t})$. This result is again in the form of **Eqn. 3** with $k_{\text{ob}} = k_{\text{ob,on}} + k_{\text{off}}$. Fitting the experimental $F_1(t)$ at each mAb concentration to **Eqn. 3** (**Figure 4c**) thus yielded k_{ob} that corresponded to $k_{\text{ob,on}} + k_{\text{off}} = k_{\text{on}}[\text{mAb}] + k_{\text{off}}$. Plotting the fitted k_{ob} as a function of the mAb concentration thus showed a linear trend, to which a linear fit gave k_{on} from the slope and k_{off} from the y-intercept (**Figure 4d**).

Simulation of displacement data. To evaluate the expected standard errors of component fractions at varying temporal resolutions (**Figure 1f**), single-molecule displacements were simulated according to the experimental conditions in **Figures 1c** and **1e**. Single-molecule displacements were simulated at a time separation of $\Delta t = 0.6$ ms for equal amounts of IgG-Cy3B and Fab-Cy3B at 12 displacements per frame pair. Of these, 46.9% were based on the diffusion mode of IgG-Cy3B ($D = 44.6$ μm²/s), 46.9% were based on the diffusion mode of Fab-Cy3B ($D = 73.3$ μm²/s), and the remaining 6.2% displacements were randomly distributed to represent background events arising from mismatched single molecules. The simulated displacements were segmented by frames at different temporal resolutions, and the IgG fraction was evaluated for each segment through fitting to the above-described 2-component model (**Eqn. 2**).

Table S1. Proteins used in this work.

Fig. No	Name in text	Full name / description	Provider	Cat#
1	Fab	Donkey Fab fragment (from anti-rabbit IgG)	Jackson	711-007-003
1	IgG	Donkey IgG (anti-mouse pAb, cross-adsorbed)	Jackson	715-005-151
2	OVA	Ovalbumin from chicken egg white	Sigma	A5503
3	BSA	Bovine serum albumin	Sigma	A3059
4, 5	GFP	*Aequorea victoria* GFP His-tag recombinant protein	Invitrogen	A42613
4	Anti-GFP mAb	Anti-GFP mouse monoclonal antibody (IgG_{2a})	Invitrogen	A-11120
5	Anti-GFP pAb	Anti-GFP rabbit polyclonal antibody (IgG)	Invitrogen	A-11122

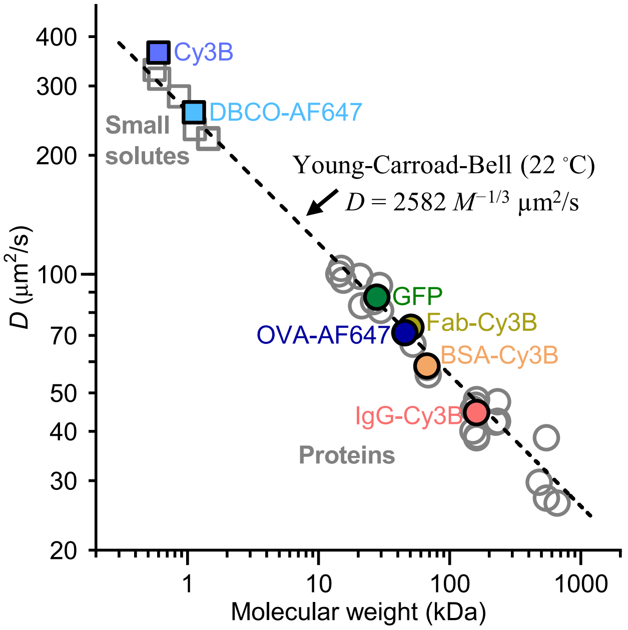

Figure S1. SM*d*M-measured diffusion coefficients *D* of different proteins (circles) and small solutes (squares) in PBS, plotted as a function of the molecular weight. Colored symbols: Molecular species examined in this study. Gray symbols: Other molecular species from previous SM*d*M results.[4,7-9] Dashed line: the Young-Carroad-Bell (YCB) model at 22 °C: D=2582·$M^{-1/3}$ μm²/s, where *M* is the molecular weight in Daltons (Da).[7]

a. IgG-Cy3B (Data in Figure 1a)

Model	Optimizing Variables	*k*	*n*	MLE results	ln(*L*)	AIC	BIC
1C	D_{1C}, b	2	31795	D_{1C}=44.6 μm²/s	-52540.0	105084.0	105100.7
2C (free D_1 ,D_2)	$D_{2C\text{-}1}$,$D_{2C\text{-}2}$,f_1, b	4	31795	D (μm²/s): 43.8 / 75.0 (0.98:0.02)	-52537.7	105083.4	105116.9

Likelihood ratio testing:

1C vs 2C ($\Delta k = 2$)

$$\Lambda = 2 \times [\ln(L_{2C}) - \ln(L_{1C})] = 2 \times (-52537.7 + 52540.0) = 4.6$$

$$p = P[\chi^2(\Delta k) > \Lambda] = P[\chi^2(2) > 4.6] = 0.10$$

b. Fab-Cy3B (Data in Figure 1b)

Model	Optimizing Variables	*k*	*n*	MLE results	ln(*L*)	AIC	BIC
1C	D_{1C}, b	2	110391	D_{1C}=73.3 μm²/s	-212029.1	424062.4	424081.6
2C (free D_1 ,D_2)	$D_{2C\text{-}1}$,$D_{2C\text{-}2}$,f_1, b	4	110391	D (μm²/s): 47.7 / 74.1 (0.02:0.98)	-212027.3	424062.7	424101.1

Likelihood ratio testing:

1C vs 2C ($\Delta k = 2$)

$$\Lambda = 2 \times [\ln(L_{2C}) - \ln(L_{1C})] = 2 \times (-212027.3 + 212029.1) = 3.6$$

$$p = P[\chi^2(\Delta k) > \Lambda] = P[\chi^2(2) > 3.6] = 0.16$$

c. 1:1 mixed IgG-Cy3B and Fab-Cy3B (Data in Figure 1c)

Model	Optimizing Variables	*k*	*n*	ln(*L*)	AIC	BIC
1C	D_{1C}, b	2	70701	-125952.0	251908.1	251926.4
2C (fixed D_1 ,D_2)	f_1, b	2	70701	-125885.7	251775.4	251793.7
3C$_i$ (extra Free D_3)	$D_{3C\text{-}3}$, f_1, f_3, b ($D_{3C\text{-}3}$ <$D_{3C\text{-}1}$ & $D_{3C\text{-}2}$)	4	70701	-125884.4	251776.8	251813.4
3C$_{ii}$ (extra Free D_3)	$D_{3C\text{-}3}$, f_1, f_3, b ($D_{3C\text{-}3}$ > $D_{3C\text{-}1}$ & $D_{3C\text{-}2}$)	4	70701	-125885.5	251779.0	251815.7

Likelihood ratio testing:

2C vs 3C$_i$ ($\Delta k = 2$)

$$\Lambda_1 = 2 \times [\ln(L_{3Ci}) - \ln(L_{2C})] = 2 \times (-125884.4+125885.7) = 2.6$$

$$p_1 = P[\chi^2(\Delta k) > \Lambda_1] = P[\chi^2(2) > 2.6] = 0.27$$

2C vs 3C$_{ii}$ ($\Delta k = 2$)

$$\Lambda_2 = 2 \times [\ln(L_{3Cii}) - \ln(L_{2C})] = 2 \times (-125885.5+125885.7) = 0.4$$

$$p_2 = P[\chi^2(\Delta k) > \Lambda_2] = P[\chi^2(2) > 0.4] = 0.82$$

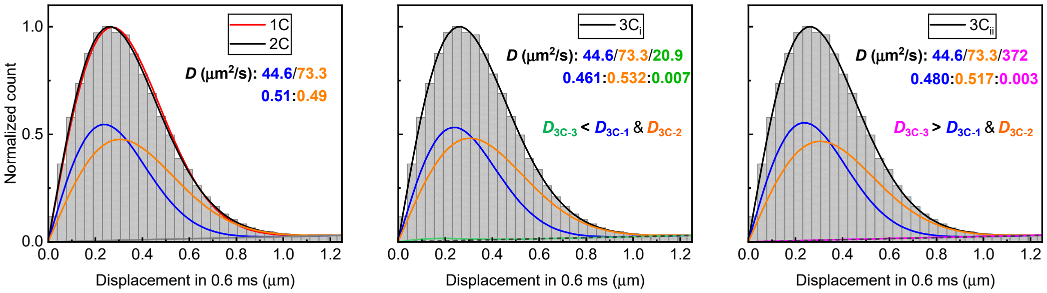

Figure S2. Model-order selection for SM*d*M single-molecule displacements. (**a,b**) Comparison of single-component (1C) and two-component (2C) models to the distributions of SM*d*M-measured 0.6-ms single-molecule displacements of IgG-Cy3B (**a**) and Fab-Cy3B (**b**) in PBS as shown in Figure 1ab. While the 2C model yields AIC (Akaike information criterion) values comparable to the 1C model, the substantial increases in BIC (Bayesian information criterion) provide strong evidence against the additional parameters, indicating that the 2C model is not justified. Consistently, likelihood ratio tests do not support the more complex model ($p = 0.10$ and 0.16, respectively). Together, both the IgG-Cy3B and Fab-Cy3B SM*d*M data are best modeled as 1C. (**c**) Comparison of single-component (1C), two-component (2C), and three-component (3C) fits to the distribution of SM*d*M-measured 0.6-ms single-molecule displacements of the 1:1 mixture of IgG-Cy3B and Fab-Cy3B as shown in Figure 1c. MLE results are shown in the plots. The 2C model based on the 1C-determined *D* values of IgG-Cy3B and Fab-Cy3B significantly improves the fit over the 1C model. Introducing a third component only marginally increases the likelihood with $p = 0.27$ and 0.82 for the two tested configurations. Both AIC and BIC also identify the 2C model as being sufficient to describe the SM*d*M data measured from the binary mixture of IgG-Cy3B and Fab-Cy3B.

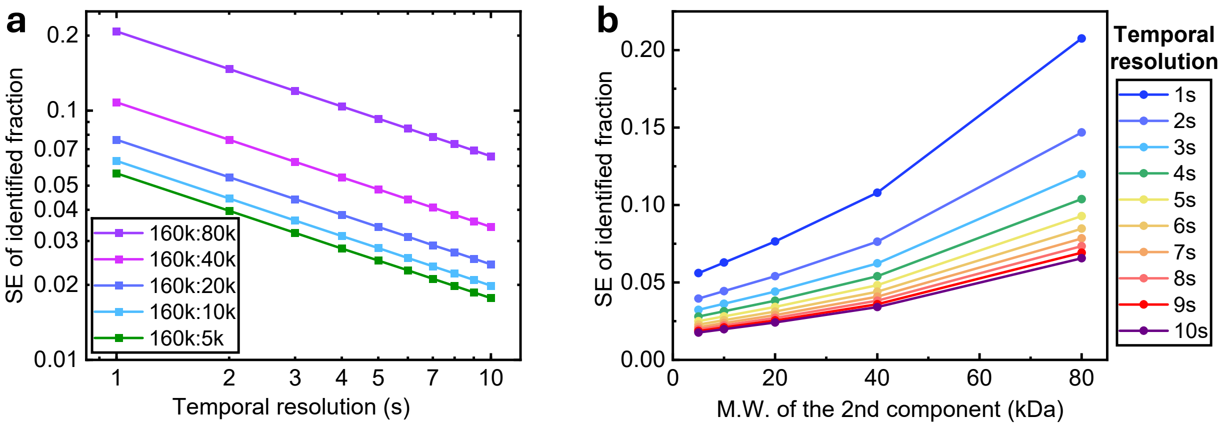

Figure S3. Expected standard errors of the SM*d*M-determined fractions for two-component mixtures of varied differences in molecular weight. Simulations were performed by holding one component at 160 kDa while varying the molecular weight of the second component. The expected *D* values of the two components were calculated based on the YCB model (Figure S1), which were used to simulate single-molecule displacements for SM*d*M two-component analysis. (**a**) Standard errors as a function of temporal resolution for varied molecular weights of the second component. (**b**) Standard errors as a function of the molecular weight of the second component (when the first component is fixed at 160 kDa) at different temporal resolutions.

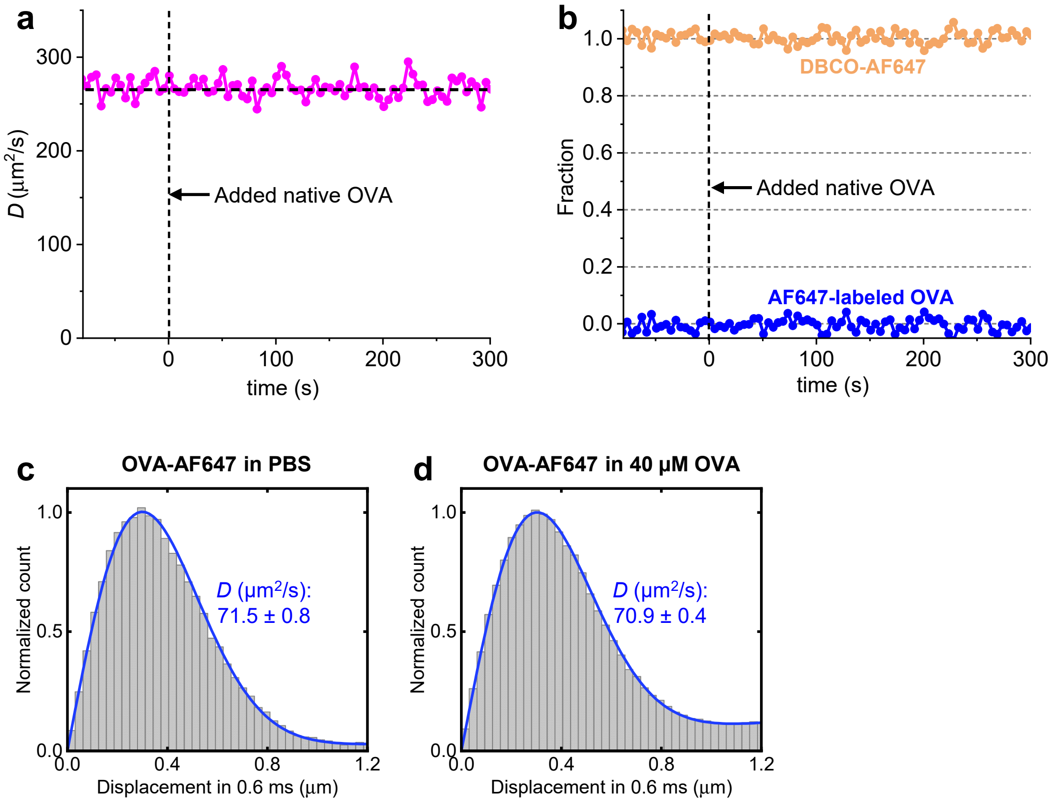

Figure S4. Control experiments for the SM*d*M kinetic study of SPAAC. (**a,b**) SM*d*M of DBCO-AF647 with the addition of unfunctionalized OVA. (**a**) SM*d*M-determined time-dependent D (based on a single-component fit) for 100 pM DBCO-AF647 in PBS, with 10 μM unfunctionalized OVA added at time 0, showing no change over time. (**b**) Fractions of DBCO-AF647 (orange) and OVA-AF647 (blue) of the detected AF647 molecules as extracted from two-component analysis of the SM*d*M data, showing no formation of the latter. (**c,d**) SM*d*M of OVA-AF647 in PBS (**c**) vs. in PBS with the addition of 40 μM OVA (**d**). Histograms: Distributions of SM*d*M-measured 0.6-ms single-molecule displacements. Curves: Fits to a single-component diffusion model, yielding $D = 71.5 \pm 0.8$ and 70.9 ± 0.4 μm²/s, respectively.

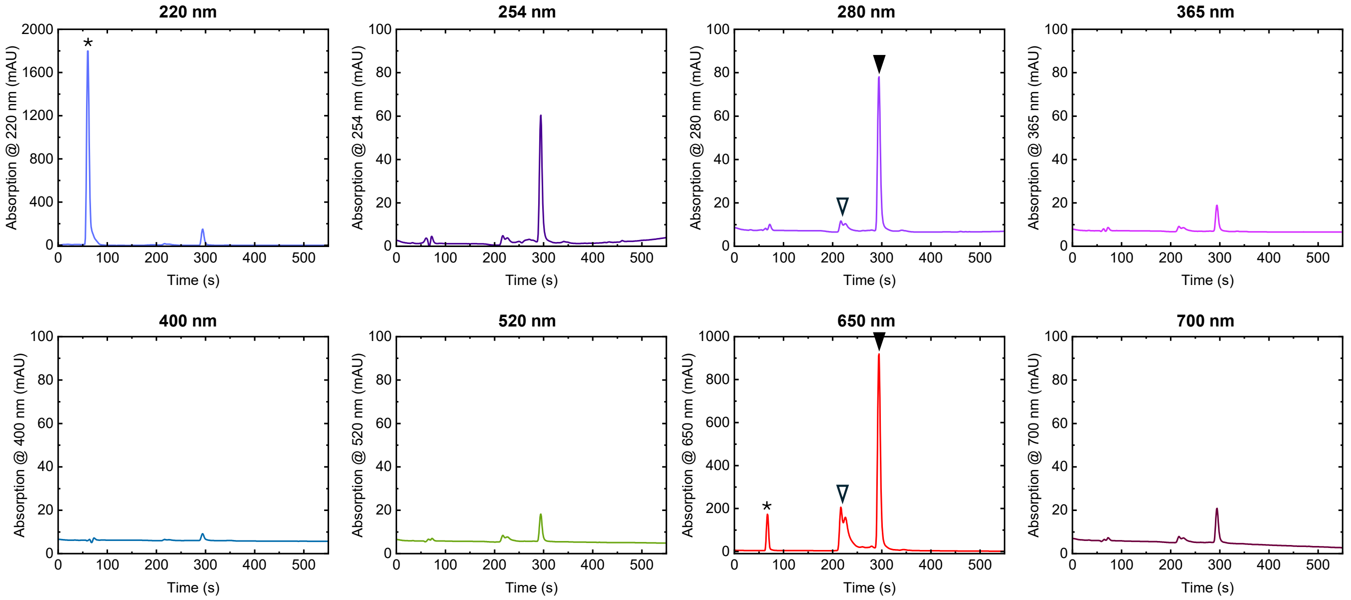

Figure S5. High-performance liquid chromatography (HPLC) analysis of the commercial DBCO-AF647 used in this study. HPLC was performed on an Agilent Infinity 1200 analytical instrument. The column used was Phenomenex Luna 5 µm C18(2) (4.6 mm I.D. × 150 mm) with a flow rate of 1.0 mL/min. DBCO-AF647 was dissolved in DMSO at 9 mM and diluted 20-fold in acetonitrile for injection. The mobile phase consisted of water with 0.05% trifluoroacetic acid (TFA) (eluent A) and acetonitrile with 0.05% TFA (eluent B), with a gradient from 10% to 100% eluent B over 10 min. Signals were monitored at 220, 254, 280, 365, 400, 520, 650, and 700 nm, as plotted above. Asterisk: Injection peak. Peaks indicated by solid and open arrow heads are designated as DBCO-AF647 and non-reactive AF647, respectively. Both species strongly absorb 650 nm, characteristic of AF647. The former shows a strong 280-nm absorption ascribed to DBCO, whereas the latter has a shorter retention time, potentially corresponding to the highly hydrophilic AF647 free dye.

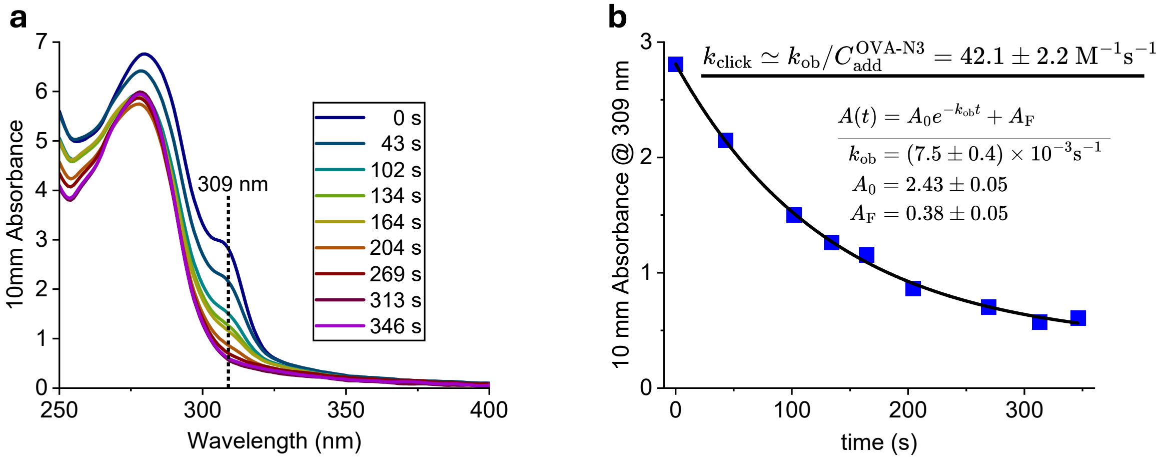

Figure S6. Estimating the reaction rate between DBCO-PEG-Biotin and OVA-N_3 through UV-Vis spectroscopy. Whereas it is difficult to detect the SPAAC product, the consumption of DBCO may be monitored from a moderate absorbance peak at 309 nm (ε = 12,000 $M^{-1}cm^{-1}$). To avoid interference from dye absorption at 309 nm, DBCO-PEG4-biotin was used instead of DBCO-AF647 used in the SM*d*M studies. 205 μM DBCO-PEG4-Biotin (Sigma 760749) was mixed with 187 μM OVA-N_3 (as described above) in PBS at t = 0. Each OVA-N_3 molecule carried ~2.7 azide groups, so the effective –N_3 concentration was >2-fold of DBCO-PEG4-Biotin. (**a**) UV-Vis spectra of the reacting solution at different time points. The DBCO absorption peak at 309 nm decreased gradually due to the SPAAC reaction. (**b**) Absorbance at 309 nm as a function of time (data points), fitted with a single-exponential decay (curve) to extract the observed first-order rate constant k_{ob} = (7.5 ± 0.4)×10^{-3} s^{-1}. The second-order rate constant is estimated by dividing k_{ob} by the initial OVA-N_3 concentration as k_{click} = (42.1 ± 2.2) $M^{-1}s^{-1}$ (between DBCO-PEG-Biotin and the ~2.7-azide-functionalized OVA-N_3).

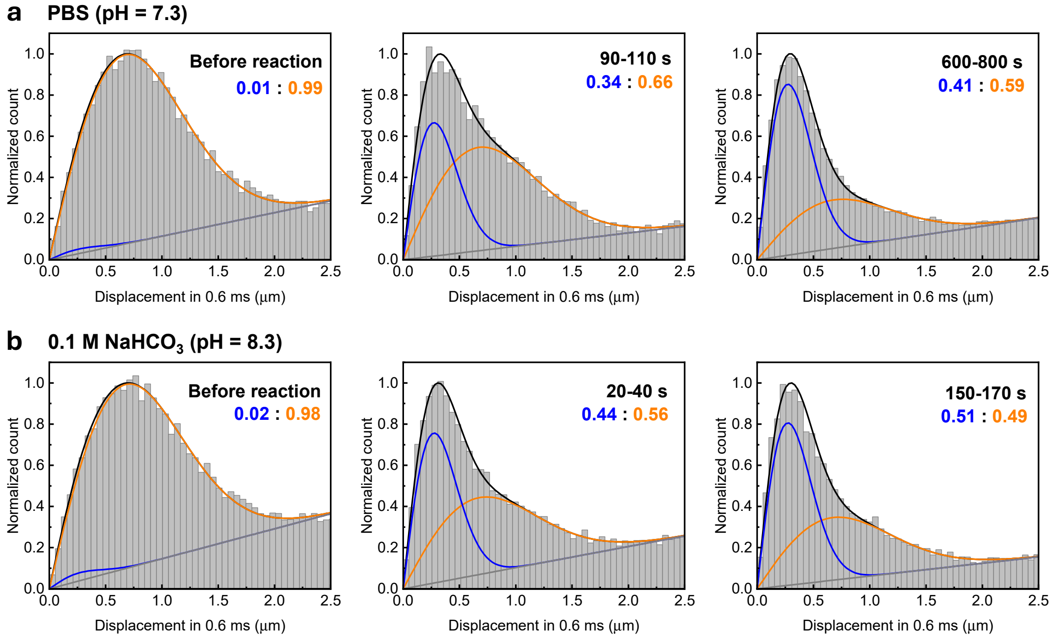

Figure S7. Two-component fitting of SM*d*M single-molecule displacements for the reaction of Cy3B-NHS ester with BSA. (**a,b**) Representative results for 100 pM Cy3B-NHS ester in PBS (**a**) or 0.1 M $NaHCO_3$ (**b**), with 20 μM BSA added at time 0. Histograms: Distributions of SM*d*M-measured 0.6-ms single-molecule displacements at the timepoints indicated in each panel. Curves: Fits to a two-component diffusion model (black) and their decomposition into BSA-Cy3B (blue) and unbound Cy3B (orange) contributions on top of single-molecule backgrounds (gray). Fitting is based on the separately determined D values of purified BSA-Cy3B at 58.5 μm²/s and unbound Cy3B at 365 μm²/s. The resulting BSA-Cy3B and unbound Cy3B fractions are labeled in each plot.

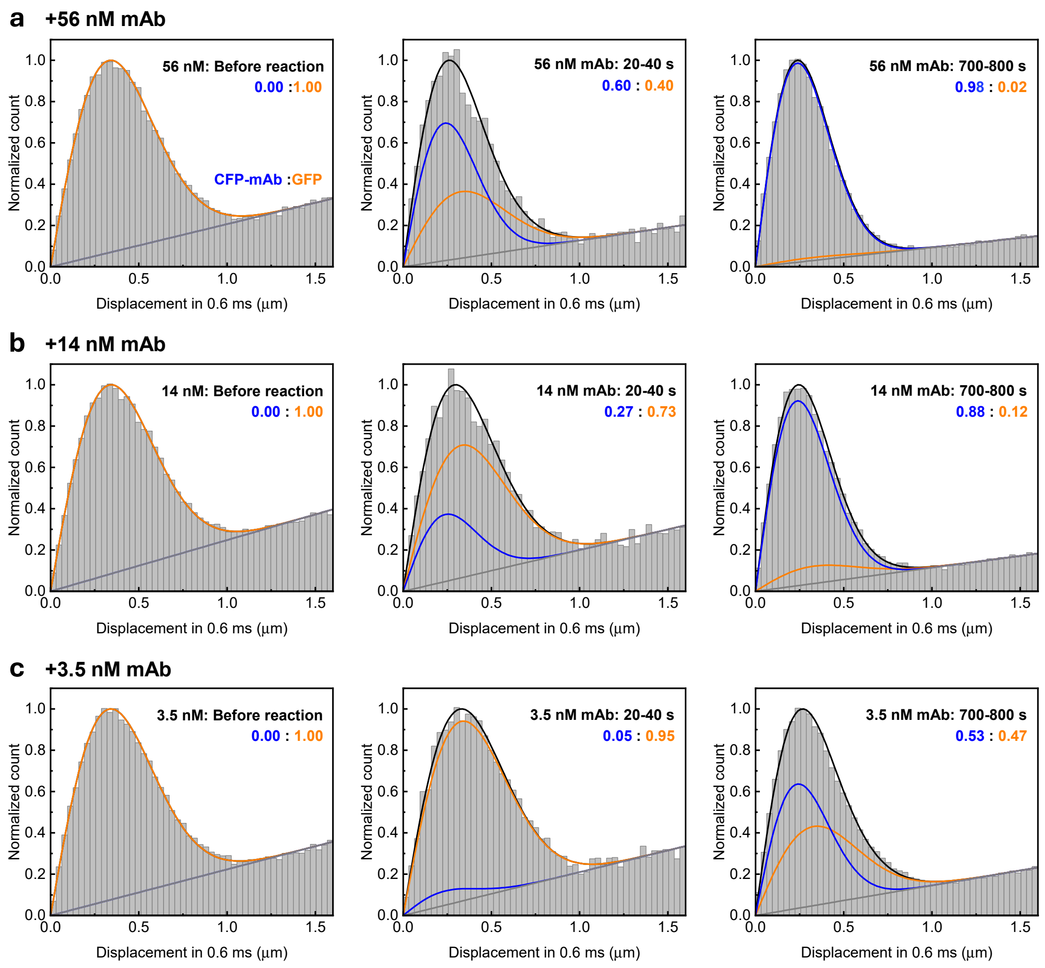

Figure S8. Two-component fitting of SM*d*M single-molecule displacements for GFP-mAb interactions. (**a-c**) Representative results for 1.2 nM GFP in PBS (1 mg/mL BSA), with 56 nM (**a**), 14 nM (**b**), and 3.5 nM (**c**) anti-GFP mAb added at time 0. Histograms: Distributions of SM*d*M-measured 0.6-ms single-molecule displacements before adding the mAb (**left**), 20-40 s after adding the mAb (**center**), and 700-800 s after adding the mAb (**right**). Curves: Fits to a two-component diffusion model (black) and their decomposition into GFP-mAb (blue) and free GFP (orange) contributions on top of single-molecule backgrounds (gray). Fitting is based on the separately determined *D* values of GFP-mAb at 44.9 μm²/s and unbound GFP at 86.4 μm²/s. The resulting GFP-mAb and free GFP fractions are labeled in each plot.

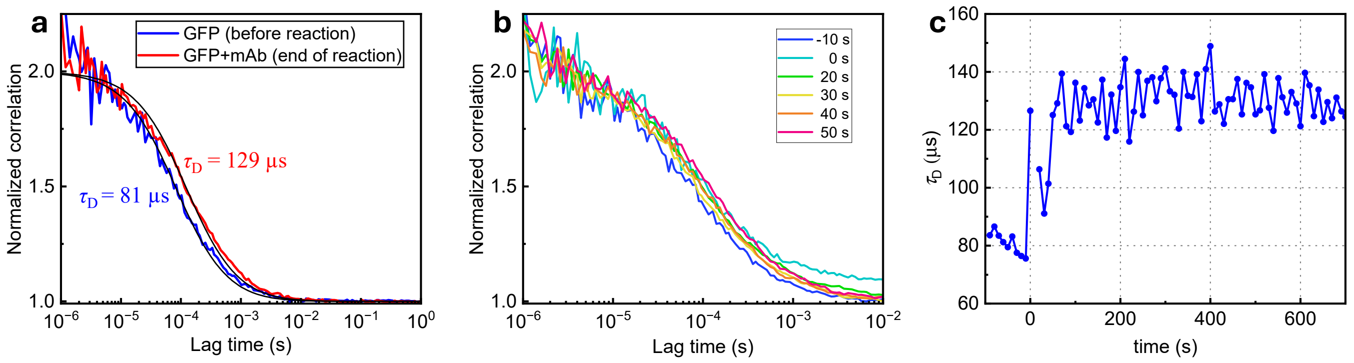

Figure S9. Fluorescence correlation spectroscopy (FCS) examination of GFP-mAb interactions. FCS was performed for 6 nM GFP in PBS (1 mg/mL BSA) on a Zeiss LSM 980 NLO Airyscan 2 system using standard settings. (**a**) FCS autocorrelation curves measured in PBS (1 mg/mL BSA) (blue), and after addition of 50 nM anti-GFP mAb (red). Black lines: Fitting based on a single diffusing species, yielding diffusion correlation time τ_D of 81 ± 4 μs and 129 ± 6 μs, respectively. (**b,c**) Monitoring the GFP-mAb interaction kinetics. FCS was performed repeatedly every 10 s for 6 nM GFP in PBS (1 mg/mL BSA), and 50 nM mAb was added. Time 0 is taken as the first timepoint showing a change in the FCS signal; the immediately following timepoint was excluded due to disturbance from sample mixing. (**b**) Autocorrelation curves right before and after mAb addition. (**c**) Fitted τ_D as a function of time. These results show that FCS detects a change in diffusion upon mAb binding, consistent with the formation of a larger complex. However, the signal-to-uncertainty ratio is substantially lower than that obtained with SMdM (Figure 4a). Moreover, the change in τ_D (and hence *D*) gives a ratio of 129/81 = 1.59 after mAb addition, translating to a 4.04-fold change in molecular weight based on the YCB model. With GFP's ~28 kDa starting molecular weight, the estimated apparent molecular weight of the product is ~110 kDa, smaller than the expected value of ~184 kDa for the GFP-mAb complex, which is more accurately captured by SM*d*M (Figure 4a,b). The underestimation of change in τ_D may arise from photophysical effects of GFP,[10] which strongly interfere with FCS autocorrelation (as suggested by the imperfect agreement of the single-component fit) but less so with single-molecule tracking.[11] Together, these results illustrate that while FCS can qualitatively detect diffusion changes, quantitative extraction of molecular size or reaction progress in such systems can be challenging.

References for the supplement

(1) He, C.; Wu, C. Y.; Li, W.; Xu, K. Multidimensional super-resolution microscopy unveils nanoscale surface aggregates in the aging of FUS condensates. *J. Am. Chem. Soc.* **2023**, *145*, 24240-24248.

(2) Moon, S.; Li, W.; Hauser, M.; Xu, K. Graphene-enabled, spatially controlled electroporation of adherent cells for live-cell super-resolution microscopy. *ACS Nano* **2020**, *14*, 5609-5617.

(3) Xiang, L.; Chen, K.; Yan, R.; Li, W.; Xu, K. Single-molecule displacement mapping unveils nanoscale heterogeneities in intracellular diffusivity. *Nat. Methods* **2020**, *17*, 524-530.

(4) Choi, A. A.; Park, H. H.; Chen, K.; Yan, R.; Li, W.; Xu, K. Displacement statistics of unhindered single molecules show no enhanced diffusion in enzymatic reactions. *J. Am. Chem. Soc.* **2022**, *144*, 4839-4844.

(5) Etheridge, T. J.; Carr, A. M.; Herbert, A. D. GDSC SMLM: Single-molecule localisation microscopy software for ImageJ. *Wellcome Open Res* **2022**, *7*, 241.

(6) Kuriyan, J.; Konforti, B.; Wemmer, D. *The molecules of life : physical and chemical principles*; Garland Science: New York, 2013.

(7) Choi, A. A.; Xiang, L.; Li, W.; Xu, K. Single-molecule displacement mapping indicates unhindered intracellular diffusion of small (≲1 kDa) solutes. *J. Am. Chem. Soc.* **2023**, *145*, 8510-8516.

(8) Li, W.; Xu, K. Super-resolution mapping and quantification of molecular diffusion via single-molecule displacement/diffusivity mapping (SM*d*M). *Acc. Chem. Res.* **2025**, *58*, 1224-1235.

(9) Choi, A. A.; Zhou, C. Y.; Tabo, A.; Heald, R.; Xu, K. Single-molecule diffusivity quantification in *Xenopus* egg extracts elucidates physicochemical properties of the cytoplasm. *Proc. Natl. Acad. Sci. U.S.A.* **2024**, *121*, e2411402121.

(10) Widengren, J.; Mets, Ü.; Rigler, R. Photodynamic properties of green fluorescent proteins investigated by fluorescence correlation spectroscopy. *Chemical Physics* **1999**, *250*, 171-186.

(11) Chen, Z.; Shaw, A.; Wilson, H.; Woringer, M.; Darzacq, X.; Marqusee, S.; Wang, Q.; Bustamante, C. Single-molecule diffusometry reveals no catalysis-induced diffusion enhancement of alkaline phosphatase as proposed by FCS experiments. *Proc. Natl. Acad. Sci. U. S. A.* **2020**, *117*, 21328-21335.
