# Supplementary material for: In Situ Kinetics of Solution-Phase Biomolecular Reactions and Interactions through Single-Molecule Displacement Statistics
Source: ACS Cent Sci. 2026 May 5;12(5):712–8. doi: 10.1021/acscentsci.6c00063 (PMC13220185; doi:10.1021/acscentsci.6c00063)
Supplement: Supplementary file 2 [file oc6c00063_si_002.pdf]

oc-2026-00063t.R1

Name: Peer Review Information for "In situ kinetics of solution-phase biomolecular reactions and interactions through single-molecule displacement statistics"

First Round of Reviewer Comments

Reviewer: 1

Comments to the Author

Li and Xu measure three distinct samples that undergo a large diffusion constant change upon binding or reacting using their unique single molecule displacement microscopy (SMdM). The conclusions are strongly supported by the data and the presentation is clear. While interesting, the application is limited to low concentration labeled reactants (~100 pM, fluorescence bioconjugation reactions). Further, without comparison to other established methods to measure diffusion constant changes, the potential impact of the work for the broader field of chemistry is not demonstrated. Therefore, while technically sounds and interesting, the work should be published in a more specialized journal, such as The Journal of Physical Chemistry B with minor revisions, unless more extensive experiments beyond SMdM are performed.

Major comments:

Comparison to current methods such as fluorescence correlation spectroscopy or dynamic light scattering would make a more convincing argument to support these results for publication in ACS Central Science. While there is a brief mention on Page 2 in the introduction, an apples-to-apples comparison of the same sample with the author’s method and a commercial FCS or DLS would be demonstrate SMdM’s place as a characterization tool. DLS is particularly relevant for the antibody sample and the use of the technology in the biopharmaceutical industry.

The D values are reported without a measure of precision – all numbers should include a report of the precision/uncertainty. Further, what is the sensitivity of the method – given the current significant figures reported, the numbers imply a difference of 0.01 um^2/s could be resolved. Is that true?

Minor comments:

It would be helpful to include more details of how the two component fit was performed in the main text and include the SMdM distibutions in Figures 3 and 4. While this is a routine method for the authors, it is quite specialized for readers that including this data would be a helpful connection between the measured observations and kinetic results. It is unclear in the main text if the fit is blind to D or if users supply an input for D and/or fraction of the population.

The emphasis on an “unlabeled reactant” (Page 1, Line 31) is a bit overstated since one part of the reaction still requires a label. A comparison to other partially unlabeled single molecule methods, such as COMPEITS (DOI: 10.1038/s41557-019-0288-8), may be worth mentioning.

What is meant by “minimal sample requirement” mentioned both in the abstract and conclusion? The amount of solution needed? This probably is very similar to the amount of solution needed for FCS or DLS requirements. But the instrumentation and analysis requirements for SMdM are much more extensive.

Page 8, line 22: why did the BSA concentration line not pass through the origin? While mentioned a physical reason is not discussed or explored.

Figure 4f – the differences in the histogram distributions are quite subtle and could be greatly effected by user-selected bin size. Plotting data on a cumulative distribution that removes the artifacts from histogram binning is highly suggested (see, DOI: 10.1039/C2AN16219A).

Reviewer: 2

Comments to the Author

This manuscript presents a clever extension of SMdM, a method developed by the PI, to examine reaction kinetics in which the labeled reactant will undergo a large increase in molecular weight, and thus a significant decrease in diffusion coefficient, upon forming product. The diffusion coefficient is quantified by measuring single-molecule displacement in paired images in which stroboscopic imaging enables sampling fluorescent molecule position in tightly spaced time intervals, allowing determination of fast diffusion coefficients in aqueous solution. The authors successfully tracked reaction progress and demonstrated across three different systems. The idea is clever, the study is well carried out, and the results are significant and of broad interest to the chemistry community, and therefore suitable for ACS Central Science. I recommend publication, after the authors take into account the following comments in preparing a revision.

1. Although the measurements are single-molecule-based detection, it requires ensemble sampling, i.e., many displacements from many molecules, instead of watching a single molecule undergoing reaction. This should be clearly stated in abstract/introduction, etc.

2. Broad applicability is advocated in the writing, but the authors should acknowledge the limitations:

a. Fluorescent labeling is required, or naturally fluorescent species is needed (e.g., GFP).

b. One of the reactants must be a macromolecule, so that the labeled reactant, upon reacting, can have a large molecular weight change to result in a significant change in diffusion coefficient.

3. Resolution limit. The method relies on fitting displacement distributions to a two-component model (e.g., reactant vs. product), with large molecular weight changes. It would be beneficial for the authors to comment on the resolution limits of the method. Figure S1 shows the relationship between D and molecular weights, but it is difficult to gauge how large the difference in diffusion coefficients (or molecular weights) must be to reliably distinguish two components.

4. The authors did not present a comparison between one-state, two-state, and three-state models. If the data were fitted using maximum likelihood estimation (MLE), it would

be appropriate to perform a likelihood ratio test to formally compare models, or to evaluate model selection criteria such as AIC or BIC.

5. The rationale for selecting the 0.6 ms time interval is not stated.

6. Viscosity effects at high concentrations. The authors add unlabeled reactants at concentrations up to 40 μM (OVA-N3) or higher. Could such additions affect the macroscopic viscosity of the solution and thereby shift the baseline D? This could complicate the two-component fit, which fixes D values based on pure component measurements. A brief clarification or control would strengthen the analysis.

7. The ~18% non-reactive dye in DBCO-AF647. The authors note that reactions with 20 and 40 μM OVA-N_3 plateau at ~0.82 OVA-AF647 fraction, attributing the remainder to non-reactive dye. This is a significant fraction. Was this verified independently (e.g., by mass spectrometry or HPLC of the starting material)? If this is a common issue with commercial DBCO-AF647, it should be noted. If not verified, the possibility that this plateau reflects an equilibrium or incomplete reaction should be discussed.

8. Polyclonal antibody aggregation. The authors observe that pAb leads to large cross-linked complexes and support this with data showing increased single-particle brightness (Figure 5c), suggesting multiple fluorophores per particle. However, the interpretation assumes that GFP fluorescence is not quenched upon antibody binding and that the detection efficiency is independent of complex size. These assumptions should be explicitly stated and, ideally, verified. Have the authors considered a simple orthogonal validation, e.g., dynamic light scattering or size-exclusion chromatography, to confirm the formation of these large aggregates?

9. Figure 2c: for the 5 μM OVA-N_3 condition, the reaction progress has clearly not plateaued by 1200 s, yet the next (and final) data point jumps to 3.5 hr. The large gap makes it difficult to assess whether the first-order kinetic fit is adequate for this condition. Intermediate time points or a discussion of the fitting reliability at low conversion would strengthen this analysis.

10. BSA background in antibody experiments. The authors add 1 mg/mL BSA to the antibody binding experiments but do not explain why. Presumably this is to prevent nonspecific adsorption, but this should be stated. Does BSA contribute to the single-molecule background? Could BSA-GFP interactions (as discussed in Ref. 30 from the same group) affect the measured D of free GFP?

11. The equilibrium dissociation constant is denoted as kD in the text (e.g., kD = 2.0 ± 0.3 nM), but the standard notation is KD (capital K) to distinguish it from rate constants.

12. The use of the YCB model to convert D to molecular weight is central to the interpretation. While Figure S1 presents the D vs. molecular weight relationship with the YCB model, it would be helpful to explicitly include the linear equation together with the uncertainties, so that readers can assess the reliability of the absolute molecular weights reported in the main text.

Author's Response to Peer Review Comments:

Response to Reviewers

Reviewer: 1

Recommendation: Publish elsewhere Journal of Physical Chemistry B

Comments:

Li and Xu measure three distinct samples that undergo a large diffusion constant change upon binding or reacting using their unique single molecule displacement microscopy (SMdM). The conclusions are strongly supported by the data and the presentation is clear. While interesting, the application is limited to low concentration labeled reactants (~100 pM, fluorescence bioconjugation reactions). Further, without comparison to other established methods to measure diffusion constant changes, the potential impact of the work for the broader field of chemistry is not demonstrated. Therefore, while technically sounds and interesting, the work should be published in a more specialized journal, such as The Journal of Physical Chemistry B with minor revisions, unless more extensive experiments beyond SMdM are performed.

Response: We thank the reviewer for their thoughtful summary and analysis. Please see our response below. For the discussion above, we note that our measurements benefit from using a very low concentration of the fluorescently labeled reactant, while a second, unlabeled reactant can be added over a wide concentration range, which enables facile kinetic analysis under pseudo-first-order conditions.

Major comments:

Comparison to current methods such as fluorescence correlation spectroscopy or dynamic light scattering would make a more convincing argument to support these results for publication in ACS Central Science. While there is a brief mention on Page 2 in the introduction, an apples-to-apples comparison of the same sample with the author's method and a commercial FCS or DLS would be demonstrate SMdM's place as a characterization tool. DLS is particularly relevant for the antibody sample and the use of the technology in the biopharmaceutical industry.

Response: Thank you for this thoughtful discussion. Although DLS has been powerful for particle sizing, its applicability to the systems studied here is limited by several factors. DLS signals scale strongly with particle size, making it challenging to detect small molecules such as DBCO-AF647 and NHS ester. Meanwhile, our GFP-antibody experiments were performed in the presence of 1 mg/mL BSA, which would dominate the DLS signal. Moreover, even for large proteins like IgG, the recommended working concentration for DLS is still 1-10 mg/mL, much higher than the nM (~0.1 µg/mL) IgG concentrations used in typical antibody-binding assays.

Meanwhile, FCS may be more meaningfully compared with our results. However, FCS has known challenges for quantitative particle sizing, as discussed in the recent review we cited (*Biophys. J.* 124, 3319). In particular, FCS is susceptible to photophysical effects, which may complicate results in reacting systems. As a notable example, FCS reported a >30% increase in the diffusion coefficient (*D*) for enzymes in catalytic reactions (*Nature* 517, 227). However, a follow-up study by the same lab showed that this *D* enhancement was an artifact due to reactant-induced photophysics, which vanished with single-molecule tracking (*PNAS* 117, 21328), in agreement with our SM*d*M results (*JACS* 144, 4839).

In response to the reviewer's suggestion, we have performed FCS measurements on the GFP–mAb system for direct comparison. At a higher GFP concentration of 6 nM, we were able to observe an increase in the diffusion correlation time τ_D upon anti-GFP mAb application, but with limited sensitivity. Moreover, the measurement underestimated the change in molecular weight, likely due to GFP photophysics. Overall, FCS remains difficult to apply as a quantitative tool for molecule sizing or reaction dynamics. In contrast, SM*d*M enables absolute *D* quantification, molecular-weight determination, and extraction of population fractions though high-throughput single-molecule displacement statistics. <u>We have added these new results and discussions to new Figure S9.</u>

The D values are reported without a measure of precision – all numbers should include a report of the precision/uncertainty. Further, what is the sensitivity of the method – given the current significant figures reported, the numbers imply a difference of 0.01 um^2/s could be resolved. Is that true?

Response: Thank you for this discussion. We have previously shown that the relative uncertainty of *D* (σ_D/D) scales inversely with the square root of the number of single-molecule displacements detected (*JACS* 144, 4839). Under our typical experimental conditions, better than ±2% uncertainty in *D* is achieved with just 10 s of SM*d*M data, and the uncertainty improves further with longer recordings. For slow diffusion, *e.g.*, *D* ~4 μm^2/s in lipid membranes, this indeed translates to ~0.01 μm^2/s uncertainties, which we utilized in our recent work to show anesthetic-enhanced diffusion (*ACS Nano* 19, 39864). We have added *D* uncertainties to the text. We also added a discussion (Page 4, Paragraph 1): "the relative uncertainties in *D* scale inversely with the square root of the number of single-molecule displacements detected.[29]"

Minor comments:

It would be helpful to include more details of how the two component fit was performed in the main text and include the SMdM distibutions in Figures 3 and 4. While this is a routine method for the authors, it is quite specialized for readers that including this data would be a helpful connection between the measured observations and kinetic results. It is unclear in the main text if the fit is blind to D or if users supply an input for D and/or fraction of the population.

Response: Thank you for this comment. Our two-component analysis uses fixed *D* values determined from pure reactants and products, and fits for the reactant and product fractions. We have improved our description in the text, *e.g.*, "fitting the SM*d*M displacement distributions with a two-component model that utilized the above *D* values of pure IgG-Cy3B and Fab-Cy3B", "we applied a two-component analysis (Figure S7) using the above-determined *D* values of GFP-mAb and unbound GFP…". We have also added two-component fitting examples of SM*d*M distributions in Figures 3 and 4 as new Figures S7 and S8.

The emphasis on an "unlabeled reactant" (Page 1, Line 31) is a bit overstated since one part of the reaction still requires a label. A comparison to other partially unlabeled single molecule methods, such as COMPEITS (DOI: 10.1038/s41557-019-0288-8), may be worth mentioning.

Response: Thank you for this comment. We note that the original sentence "Working with ~100 pM fluorescently tagged reactant while varying the concentration of a second, unlabeled reactant over wide ranges" already indicates the use of a labeled species. Our intention was to emphasize that the concentration of the unlabeled reactant can be varied over wide ranges to extract reaction rate constants. To further emphasize the use of fluorescent labels, we have added to the Conclusions "provided that one of the reactants is fluorescently labeled…". COMPEITS is indeed an elegant approach, but it is meant for surface reactions, where the competition for finite surface sites yields reaction kinetics for the non-fluorescent species.

What is meant by "minimal sample requirement" mentioned both in the abstract and conclusion? The amount of solution needed? This probably is very similar to the amount of solution needed for FCS or DLS requirements. But the instrumentation and analysis requirements for SMdM are much more extensive.

Response: Thank you for this discussion. Indeed the used solution amount is comparable to FCS; this discussion was meant to compare with bulk spectroscopy methods. The ~100 pM concentration of the fluorescent species is still 10-fold lower than in typical FCS experiments. SM*d*M is based on standard wide-field single-molecule microscopy, and our analysis, which fits single-molecule displacements to two components of MW-defined *D* values, is more intuitive than FCS and DLS correlation analysis, where many assumptions are made and absolute *D* values are difficult to obtain even after extensive calibration. We have changed "minimal sample" to "microliter-scale sample" in the text.

Page 8, line 22: why did the BSA concentration line not pass through the origin? While mentioned a physical reason is not discussed or explored.

Response: Thank you for this comment. We have added a brief discussion (Page 8, Paragraph 3): "The finite y-intercept arises from the competing hydrolysis consumption of the NHS ester, which occurs even in the absence of the aminolysis substrate BSA. Simple derivations (**Methods**) showed that the slope of the fit gave the second-order rate constant of aminolysis, $k_{Aminolysis} = 291 \pm 6\ M^{-1}s^{-1}$, whereas the y-intercept gave the first-order rate constant of hydrolysis, $k_{Hydrolysis} = (2.34 \pm 0.04) \times 10^{-3}\ s^{-1}$." The actual derivations are given in the Methods section, as indicated.

Figure 4f – the differences in the histogram distributions are quite subtle and could be greatly effected by user-selected bin size. Plotting data on a cumulative distribution that removes the artifacts from histogram binning is highly suggested (see, DOI: 10.1039/C2AN16219A).

Response: Thank you for this discussion. In Figure 4f, we did not observe noticeable differences in single-particle photon counts before and after mAb addition, in contrast to our results in Figure 5c, where we observed a substantial shift towards higher photon counts after pAb addition. To improve visualization, we have replotted the distributions as curves rather than histogram bars. We have also clarified in the caption of Figure 4f that here we observe no substantial differences, in contrast to the pAb results in Figure 5c.

Additional Questions:

Quality of experimental data, technical rigor: Top 10%

Significance to chemistry researchers in this and related fields: Moderate

Broad interest to other researchers: Moderate

Novelty: Top 10%

Is this research study suitable for media coverage or a First Reactions (a News & Views piece in the journal)?: No

Reviewer: 2

Recommendation: Publish in ACS Central Science after minor revisions noted.

Comments:

This manuscript presents a clever extension of SMdM, a method developed by the PI, to examine reaction kinetics in which the labeled reactant will undergo a large increase in molecular weight, and thus a significant decrease in diffusion coefficient, upon forming product. The diffusion coefficient is quantified by measuring single-molecule displacement in paired images in which stroboscopic imaging enables sampling fluorescent molecule position in tightly spaced time intervals, allowing determination of fast diffusion coefficients in aqueous solution. The authors successfully tracked reaction progress and demonstrated across three different systems. The idea is clever, the study is well carried out, and the results are significant and of broad interest to the chemistry community, and therefore suitable for ACS Central Science. I recommend publication, after the authors take into account the following comments in preparing a revision.

Response: We thank the reviewer for their excellent summary, enthusiasm for our work, and rigorous analysis and questions. Please see our response below.

1. Although the measurements are single-molecule-based detection, it requires ensemble sampling, i.e., many displacements from many molecules, instead of watching a single molecule undergoing reaction. This should be clearly stated in abstract/introduction, etc.

Response: Thank you for this discussion. We have added these discussions to our revision. Abstract: "Fitting the time-dependent displacement distributions of many molecules…". Introduction: "By temporally segmenting the SM*d*M-accumulated displacements of many single molecules…"

2. Broad applicability is advocated in the writing, but the authors should acknowledge the limitations:

a. Fluorescent labeling is required, or naturally fluorescent species is needed (e.g., GFP).

b. One of the reactants must be a macromolecule, so that the labeled reactant, upon reacting, can have a large molecular weight change to result in a significant change in diffusion coefficient.

Response: We have added discussions on these limitations in our Conclusions: "provided that one of the reactants is fluorescently labeled and undergoes substantial changes in molecular size in the reaction". We also revised "broadly applicable across diverse reaction types" to "applicable to diverse reaction types" in the abstract and Conclusions. See further discussions related to (b) in our response to #3 below.

3. Resolution limit. The method relies on fitting displacement distributions to a two-component model (e.g., reactant vs. product), with large molecular weight changes. It would be beneficial for the authors to comment on the resolution limits of the method. Figure S1 shows the relationship between D and molecular weights, but it is difficult to gauge how large the difference in diffusion coefficients (or molecular weights) must be to reliably distinguish two components.

Response: Thank you for this thoughtful discussion. In Figure 1, we examined how well we can quantify two components ~3× different in size, namely, the 50 kDa Fab and the 160 kDa IgG, and showed, with both experimental and simulated data, standard errors of 0.05 and 0.03 for the determined component fractions at 4 s and 10 s time resolutions, respectively. Inspired by the reviewer's comment, we have added additional analysis to examine how well we can quantify two components with varied differences in molecular weight (Page 5, Paragraph 1): "Simulation further predicted increased fraction uncertainties with reduced molecular-weight differences between the two components and *vice versa*, with 2-fold and 32-fold differences yielding standard errors of 0.066 and 0.018 at a 10 s temporal resolution, respectively (**Figure S3**). If we fluorescently tag the smaller reactant in a conjugation reaction, the molecular-weight difference between the SM*d*M-detected product and reactant is >2 and easily >10 (examples below), and their fractions in the mixture should be reliably quantified at 1-10 s temporal resolution."

4. The authors did not present a comparison between one-state, two-state, and three-state models. If the data were fitted using maximum likelihood estimation (MLE), it would be appropriate to perform a likelihood ratio test to formally compare models, or to evaluate model selection criteria such as AIC or BIC.

Response: Thank you for this discussion. In our experiments, we first perform SM*d*M on the pure reactant and product, and then treat the SM*d*M-obtained single-molecule displacements at each timepoint in the reaction as a mixture of the reactant and product states with varying fractions. In this revision, we have performed new analyses as suggested by the reviewer to show that our MLE analysis of SM*d*M data can correctly identify pure molecules and binary mixtures as one and two components, respectively. We have added these results to new Figure S2.

5. The rationale for selecting the 0.6 ms time interval is not stated.

Response: For SM*d*M of free diffusion in solution, we have been using a 0.6 ms time interval, for which the moderate single-molecule displacements allow us to efficiently track molecules between paired frames. We previously compared results obtained with several time intervals, showing comparable *D* values. See

Figure S2 of *JACS* 144, 4839. We have added a discussion to the text (first paragraph in Results and Discussion): "which we previously showed to enable efficient detection of single-molecule displacements in solution.[29]"

6. Viscosity effects at high concentrations. The authors add unlabeled reactants at concentrations up to 40 µM (OVA-N3) or higher. Could such additions affect the macroscopic viscosity of the solution and thereby shift the baseline D? This could complicate the two-component fit, which fixes D values based on pure component measurements. A brief clarification or control would strengthen the analysis.

Response: Thank you for this rigorous discussion. The higher limit of 40 µM OVA-N3 in our experiments corresponds to ~0.18 wt% protein. This concentration does not yet significantly affect the solution's viscosity, as shown in the literature and by our own measurements. In this revision, we compared SM*d*M of OVA-AF647 in PBS vs. with the addition of 40 µM OVA, and found no significant difference in *D*. Moreover, since changes in solution viscosity are immediate, if this factor had affected our two-population fit, we would have observed a sudden jump in the fitted product fraction at time 0 as we added OVA-N3, but we did not observe such a jump. We have added a discussion to the text (Page 5, last paragraph): "As another control, the diffusivity of OVA-AF647 in PBS was unaffected by the addition of 40 µM OVA (**Figure S4**)."

7. The ~18% non-reactive dye in DBCO-AF647. The authors note that reactions with 20 and 40 µM OVA-N_3 plateau at ~0.82 OVA-AF647 fraction, attributing the remainder to non-reactive dye. This is a significant fraction. Was this verified independently (e.g., by mass spectrometry or HPLC of the starting material)? If this is a common issue with commercial DBCO-AF647, it should be noted. If not verified, the possibility that this plateau reflects an equilibrium or incomplete reaction should be discussed.

Response: In typical dye-conjugation applications, one adds DBCO-dye in excess and removes unconjugated dye after the reaction, so non-reactive dyes are not a concern. Following the reviewer's suggestion, we performed HPLC on our commercial DBCO-AF647 and indeed identified a significant non-reactive dye fraction. We have added this result to **Figure S5**.

8. Polyclonal antibody aggregation. The authors observe that pAb leads to large cross-linked complexes and support this with data showing increased single-particle brightness (Figure 5c), suggesting multiple fluorophores per particle. However, the interpretation assumes that GFP fluorescence is not quenched upon antibody binding and that the detection efficiency is independent of complex size. These assumptions should be explicitly stated and, ideally, verified. Have the authors considered a simple orthogonal validation, e.g., dynamic light scattering or size-exclusion chromatography, to confirm the formation of these large aggregates?

Response: Thank you for this discussion. GFP has robust fluorescence as its fluorophore is well protected by the beta barrel. We thus do not expect antibody binding/complexing to alter its brightness. For example, tandem GFPs generally show linear scaling of brightness with the number of GFPs. Prompted by the reviewer's question, we looked into this system further and found that at the macroscopic level, the cross-linking between antigens and polyclonal (but not monoclonal) antibodies is well documented as the precipitin reaction, in which visible precipitates form at high concentrations. Our very low (~1 nM) antigen (GFP) concentration led to the gradual formation of nanoscale antigen-antibody complexes, which would be difficult to capture using DLS or size-exclusion chromatography that operate at much higher concentrations. We have added these discussions to our revision (Page 11, first paragraph).

9. Figure 2c: for the 5 µM OVA-N_3 condition, the reaction progress has clearly not plateaued by 1200 s, yet the next (and final) data point jumps to 3.5 hr. The large gap makes it difficult to assess whether the first-order kinetic fit is adequate for this condition. Intermediate time points or a discussion of the fitting reliability at low conversion would strengthen this analysis.

Response: For each condition, we recorded a similar ~1300 s reaction time on the microscope, and then spot-checked at 3.5 hr for the 20 and 40 μM OVA-N_3 conditions. The SPAAC reaction here has no side or reverse reactions, and first-order kinetics should be appropriate. The low conversion in the 5 μM OVA-N_3 condition means only a small fraction of DBCO-AF647 was consumed, so we observed little change in reaction rate over time, and it is indeed more difficult to compare modes. However, k_{ob} from the first-order kinetic fit followed 2nd-order reaction well for the different OVA-N_3 concentrations, suggesting the fitting was valid.

10. BSA background in antibody experiments. The authors add 1 mg/mL BSA to the antibody binding experiments but do not explain why. Presumably this is to prevent nonspecific adsorption, but this should be stated. Does BSA contribute to the single-molecule background? Could BSA-GFP interactions (as discussed in Ref. 30 from the same group) affect the measured D of free GFP?

Response: Indeed, BSA is commonly added in antibody-binding assays to block nonspecific adsorption and interactions. Moreover, BSA is commonly added to antibodies as a stabilizer. For example, the monoclonal antibody used in our work was provided in "PBS with BSA" as the storage medium. The SM*d*M-measured GFP *D* values are consistent with YCB and previously reported values, indicating no interactions with BSA, as expected based on Ref 30, given that GFP is negatively charged. We have added a brief discussion to the text (Page 10, Paragraph 2): "addition of 1 mg/mL BSA to block nonspecific adsorption and interactions".

11. The equilibrium dissociation constant is denoted as kD in the text (e.g., kD = 2.0 ± 0.3 nM), but the standard notation is KD (capital K) to distinguish it from rate constants.

Response: Thank you for catching this issue. We have changed to K_D.

12. The use of the YCB model to convert D to molecular weight is central to the interpretation. While Figure S1 presents the D vs. molecular weight relationship with the YCB model, it would be helpful to explicitly include the linear equation together with the uncertainties, so that readers can assess the reliability of the absolute molecular weights reported in the main text.

Response: Indeed, our results generally showed good agreement with the YCB model. Some uncertainties were from the samples themselves. For example, alkaline phosphatase of different product numbers from the same provider yielded different *D* values, suggesting different states, *e.g.*, minor impurities and oligomerization. Protein folding state is another likely variable. Thus, although our *D* values agree well with YCB, the *D*-MW relationship is not an exact mapping. In the revised manuscript, we have added uncertainty ranges for molecular weights estimated from *D*.

Additional Questions:

Quality of experimental data, technical rigor: Top 10%

Significance to chemistry researchers in this and related fields: Top 10%

Broad interest to other researchers: Top 10%

Novelty: Top 10%

Is this research study suitable for media coverage or a First Reactions (a News & Views piece in the journal)?: No
